# Estimating gene-level false discovery probability improves eQTL statistical fine-mapping precision

**DOI:** 10.1093/nargab/lqad090

**Published:** 2023-10-30

**Authors:** Qingbo S Wang, Ryuya Edahiro, Ho Namkoong, Takanori Hasegawa, Yuya Shirai, Kyuto Sonehara, Japan COVID-19 Task Force, Atsushi Kumanogoh, Makoto Ishii, Ryuji Koike, Akinori Kimura, Seiya Imoto, Satoru Miyano, Seishi Ogawa, Takanori Kanai, Koichi Fukunaga, Yukinori Okada

**Affiliations:** Department of Statistical Genetics, Osaka University Graduate School of Medicine, Suita, 565-0871, Japan; Laboratory of Statistical Immunology, Immunology Frontier Research Center (WPI-IFReC), Osaka University, Suita, 565-0871, Japan; Department of Genome Informatics, Graduate School of Medicine, the University of Tokyo, Tokyo, 113-0033, Japan; Department of Statistical Genetics, Osaka University Graduate School of Medicine, Suita, 565-0871, Japan; Department of Respiratory Medicine and Clinical Immunology, Osaka University Graduate School of Medicine, Suita, 565-0871, Japan; Department of Infectious Diseases, Keio University School of Medicine, Tokyo, 160-8582, Japan; M&D Data Science Center, Tokyo Medical and Dental University, Tokyo, 113-8510, Japan; Department of Statistical Genetics, Osaka University Graduate School of Medicine, Suita, 565-0871, Japan; Department of Respiratory Medicine and Clinical Immunology, Osaka University Graduate School of Medicine, Suita, 565-0871, Japan; Department of Statistical Genetics, Osaka University Graduate School of Medicine, Suita, 565-0871, Japan; Department of Genome Informatics, Graduate School of Medicine, the University of Tokyo, Tokyo, 113-0033, Japan; Integrated Frontier Research for Medical Science Division, Institute for Open and Transdisciplinary Research Initiatives, Osaka University, Suita, 565-0871, Japan; Department of Respiratory Medicine and Clinical Immunology, Osaka University Graduate School of Medicine, Suita, 565-0871, Japan; Integrated Frontier Research for Medical Science Division, Institute for Open and Transdisciplinary Research Initiatives, Osaka University, Suita, 565-0871, Japan; Department of Immunopathology, Immunology Frontier Research Center (WPI-IFReC), Osaka University, Suita, 565-0871, Japan; Center for Infectious Disease Education and Research (CiDER), Osaka University, Suita, 565-0871, Japan; Department of Respiratory Medicine, Nagoya University Graduate School of Medicine, 65 tsurumai, Showa-ku, Nagoya, 466-8550, Japan; Health Science Research and Development Center (HeRD), Tokyo Medical and Dental University, Tokyo, 113-8510, Japan; Institute of Research, Tokyo Medical and Dental University, Tokyo, 113-8510, Japan; Division of Health Medical Intelligence, Human Genome Center, the Institute of Medical Science, the University of Tokyo, Tokyo, 108-8639, Japan; M&D Data Science Center, Tokyo Medical and Dental University, Tokyo, 113-8510, Japan; Department of Pathology and Tumor Biology, Kyoto University, Kyoto, 606-8315, Japan; Institute for the Advanced Study of Human Biology (WPI-ASHBi), Kyoto University, Kyoto, 606-8303, Japan; Department of Medicine, Center for Hematology and Regenerative Medicine, Karolinska Institute, Stockholm, 171 77, Sweden; Division of Gastroenterology and Hepatology, Department of Medicine, Keio University School of Medicine, Tokyo, 160-8582, Japan; Division of Pulmonary Medicine, Department of Medicine, Keio University School of Medicine, Tokyo, 160-8582, Japan; Department of Statistical Genetics, Osaka University Graduate School of Medicine, Suita, 565-0871, Japan; Laboratory of Statistical Immunology, Immunology Frontier Research Center (WPI-IFReC), Osaka University, Suita, 565-0871, Japan; Department of Genome Informatics, Graduate School of Medicine, the University of Tokyo, Tokyo, 113-0033, Japan; Integrated Frontier Research for Medical Science Division, Institute for Open and Transdisciplinary Research Initiatives, Osaka University, Suita, 565-0871, Japan; Center for Infectious Disease Education and Research (CiDER), Osaka University, Suita, 565-0871, Japan; Laboratory for Systems Genetics, RIKEN Center for Integrative Medical Sciences, Yokohama, 230-0045, Japan

## Abstract

Statistical fine-mapping prioritizes putative causal variants from a large number of candidate variants, and is widely used in expression quantitative loci (eQTLs) studies. In eQTL fine-mapping, the existence of causal variants for gene expression is not guaranteed, since the genetic heritability of gene expression explained by nearby (*cis-*) variants is limited. Here we introduce a refined fine-mapping algorithm, named Knockoff–Finemap combination (KFc). KFc estimates the probability that the causal variant(s) exist in the *cis-*window of a gene through construction of knockoff genotypes (i.e. a set of synthetic genotypes that resembles the original genotypes), and uses it to adjust the posterior inclusion probabilities (PIPs). Utilizing simulated gene expression data, we show that KFc results in calibrated PIP distribution with improved precision. When applied to gene expression data of 465 genotyped samples from the Japan COVID-19 Task Force (JCTF), KFc resulted in significant enrichment of a functional score as well as reporter assay hits in the top PIP bins. When combined with functional priors derived from an external fine-mapping study (GTEx), KFc resulted in a significantly higher proportion of hematopoietic trait putative causal variants in the top PIP bins. Our work presents improvements in the precision of a major fine-mapping algorithm.

## Introduction

Statistical fine-mapping is a promising approach to prioritize putative causal variants in genome-wide association studies (GWAS) (1,2). Development of scalable statistical fine-mapping methods (3,4) allowed finer interpretation of biobank-scale association studies of complex traits or other molecular phenotypes such as gene expression (5) [expression quantitative trait loci (eQTLs) (6)] often down to single variant resolution.

Fine-mapping algorithms typically assume the existence of one or a few causal variants in a locus of interest [e.g. (3,4) assign zero prior probability of ‘no causal variants’, (7–9) assign non-zero but small probability and (10) performs thresholding]. Different approaches exist to define the unit of such a ‘locus’. In a recent biobank fine-mapping study (Kanai *et al.*, https://www.medrxiv.org/content/10.1101/2021.09.03.21262975v1), a locus is defined as containing at least one variant with a genome-wide significant (*P* < 5e-8) *P*-value, and extended so that the linkage disequilibrium (LD) between the genome-wide significant variants in the locus and variants outside is low enough.

In *cis-*eQTL fine-mapping, the size of the locus is naturally defined as the *cis-*region (e.g. ± 1 Mb) based on our biological knowledge (6,11). However, different studies use different thresholds to decide on whether or not to apply fine-mapping algorithms for a gene. In other words, fine-mapping algorithms, which *apriori* assume that there is at least one causal variant in a locus (3), will be applied only if a gene is deemed as an ‘eGene’ with variable expression as a function of the genotype of its *cis-*region. For example, Kanai *et al.* previously used a genome-wide threshold (*P* < 5e-8) to decide which genes to fine-map, whereas the GTEx consortium (6,10) used a *q*-value-based gene-level false discovery rate (FDR) threshold of 0.05. We note that these thresholding approaches, although attractive, include room for improvement, in that (i) the optimal threshold, which should ideally be a function of study design and power, is not trivial and (ii) two genes with almost identical statistical significance could be treated differently (e.g. the fine-mapping algorithm would be applied to a gene with minimum *P* = 4.99e-8, but not to another gene with minimum *P* = 5.01e-8, although such small differences in significance statistics are more likely to be by chance than due to real biological differences).

**Figure 1.**
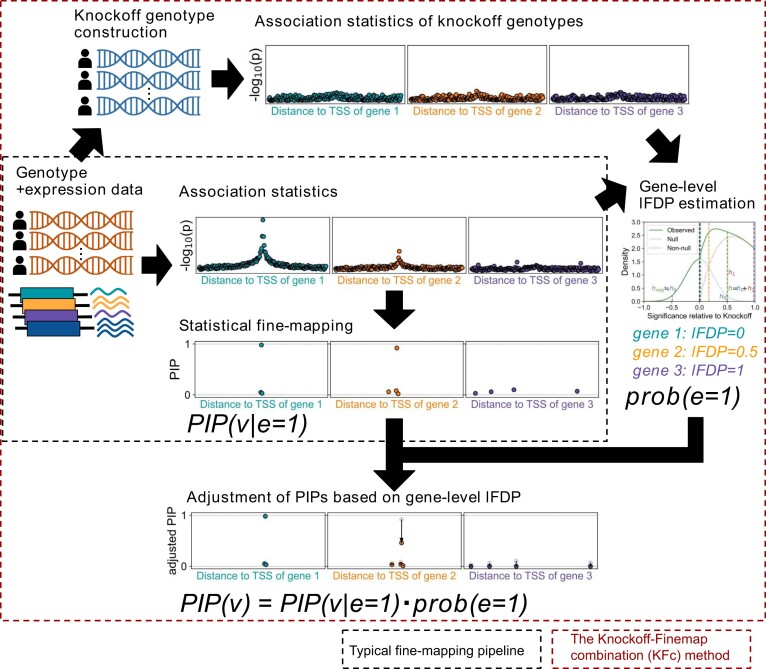
Overview of the KFc method. In contrast to a typical fine-mapping procedure where we simply apply fine-mapping algorithms after performing an association study (either with or without a *P*-value cut-off to define eGenes), we additionally calculate the FDP utilizing knockoff genotypes. We interpret raw output of statistical fine-mapping algorithms as the PIP assuming that there is a causal variant affecting the expression of the gene, and adjust the raw PIP by multiplying by (1 – FDP), reflecting the probability that there are no variants affecting the gene expression.

To investigate such thresholding problems in eQTL fine-mapping, we perform an extensive simulation of eQTL fine-mapping, using real genotype data from the Japan COVID-19 Task Force (JCTF) study of 465 samples (12), with synthetic gene expression regulation profiles and causal variant setting. Moreover, to better handle the challenges of the thresholding problems, we propose a simple augmentation of current state-of-art fine-mapping procedures, which is to first estimate the locus-level false discovery probability (FDP) for a given gene and use (1 – FDP) as a prior probability of a locus harboring at least one causal variant [which is equivalent to simply adjusting the posterior inclusion probability (PIP) by (1 – FDP) post-hoc, as will be shown in the Materials and methods] (Figure 1). For FDP estimation, we construct a knockoff genotype (13,14) (i.e. a set of synthetic genotypes that resemble the original genotypes in terms of LD structure while providing no additional predictive power for traits of interest) and utilize the symmetry of its test statistics under the null hypothesis.

After evaluating the fine-mapping performance of different thresholding methods plus the proposed Knockoff-Finemap combination (KFc) method, we apply the KFc method to the real whole-blood gene expression data from the JCTF, and suggest that the KFc method improves the quality of fine-mapping in terms of the enrichment of orthogonal functional genomics-based scores [the Expression Modifier Score = EMS (5) and the reporter assay QTLs = raQTLs (15)].

Finally, we extend our simulation framework to a hypothetical scenario of cross-population fine-mapping, and demonstrate the utility of the KFc method in the cross-population setting, in simulation as well as real data application.

## Materials and methods

### Ethics

This study was approved by the ethical committees of Keio University School of Medicine, Osaka University Graduate School of Medicine and affiliated institutes. Informed consent was obtained from all participants in the Japan COVID-19 Task Force.

### The Knockoff–Finemap combination method

The key idea of the KFc method is to explicitly consider genome-wide *cis-*eQTL fine-mapping as a three-step inference procedure, where step 1 is to estimate the probability that the causal variant(s) exist in a gene *cis-*window, step 2 is to perform *cis-*eQTL fine-mapping on a gene with an assumption that there is at least one causal variant in the *cis-*region, as is done in typical fine-mapping studies, and step 3 is to combine the information from steps 1 and 2 to derive the PIP, a credible set and other informative statistics. The novelty of the method resides in that we utilize knockoff genotype construction to derive the gene-level FDP as a continuous value in step 1, and that we realize step 3 in a simple multiplication.

Specifically, for a gene $g$, let $PIP( {v,g} )$ be the estimand, namely the probability that the variant $v$ is causal to the expression of $g$ in a tissue of interest. Our model assumes a binary property of $g$; whether *cis-*regulatory variants on $g$ exists or not (i.e. whether $g$ is an eGene or a null gene). We let $e( g ) \epsilon \{ {0,1} \}$ denote this property. From here we write $PIP( {v,g} )$ as $PIP( v )$, $e( g ) = e$ .. for simplicity (i.e. we drop the notation $g$ but remain focused on the specific gene $g$). Under the assumption, applying typical fine-mapping algorithms that assume the existence of at least one causal variant (e.g. FINEMAP or SuSiE) is equivalent to estimating $PIP(v\;|\;e = 1)$. Thus, under the model,


\begin{equation*}PIP\left( v \right) = PIP(v\;|\;e = 1) \cdot prob\left( {e = 1} \right).\end{equation*}

Estimating $prob( {e = 1} )$ (i.e. step 1) is non-trivial, where we utilize the knockoff genotype construction to estimate the local false discovery probability (lFDP), which is equivalent to $prob( {e = 0} ) = 1 - prob( {e = 1} )$. In brief, knockoff genotypes are a set of synthetic genotypes keeping the LD structure, while providing no additional predictive power for traits of interest given the real genotypes. Knockoff genotypes can thus serve as a ‘negative control’ to calibrate the estimation of $prob( {e = 1} )$. Further details of the knockoff construction are described in later sections.

KFc can thus be regarded as an extension of a typical fine-mapping pipeline that simplifies steps 1 and 3 as a binary selection applying a hard threshold (e.g. minimum *P* < 5e-8) to first define eGenes to fine-map (or no selection at all, applying fine-mapping algorithms to all the genes regardless of their marginal association statistics; in such cases $prob( {e = 1} )$ constantly). Using the notations above, binary selection corresponds to letting $prob( {e = 1} ) = 1$ if and only if the minimum *P* is < threshold (and is 0 otherwise).

We note that in reality, the binary assumption above would not hold true, which directly highlights the value of letting $prob( {e = 1} )$ be a continuous value as a function of the study sample.

The major computational burden of the method is on steps 1 (constructing knockoff genotypes) and 2 (performing fine-mapping). Convergence of each of these steps in a reasonable time when applied to a dataset of >300 000 individuals (UK Biobank) was demonstrated previously by different groups (3,14) [e.g. 72 h for step 2 (14)]. Although dependent on multiple factors such as number of variants, available resources and parameter settings, those previous studies suggest that KFc has the potential to be scaled up for biobank analyses.

### Simulation setup

Throughout the simulations in this work (as well as the real data example, as discussed in later sections), we used the genotype data of *n* = 465 samples from the JCTF study (https://humandbs.biosciencedbc.jp/en/hum0343-v2). The samples are ascertained for COVID-19 cases, and include 359 samples diagnosed as severe or most severe cases.

The samples and genetic variants have already gone through stringent quality control (QC) processes, resulting in 415 348 common (minor allele frequency > 0.01) genotyped variants, and a total of 18 343 752 variants including imputed variants.

We used 19 913 genes that are tested for eQTL association after undergoing QC in the original JCTF study, and assigned the simulated ‘expression level’ for those genes in the following steps.

- *Assigning heritability for each gene*

We fit the heritability distribution from a prior study (16) to simulate a realistic heritability distribution. We then randomly assigned the genetic heritability explained by hypothetical ‘causal’ variants for each gene, ranging from 0 to 0.99 in a step window size of 0.01.

- *Assigning causal variant(s) for each gene with non-zero heritability*

For each gene with non-zero heritability (i.e. ‘eGenes’, as opposed to ‘null genes’ with zero heritability), we assign the number of ‘causal’ variants, ranging from 1, 2, …to 5. To assign causal variant(s) in a biologically plausible way, we first utilized the existing result of statistical fine-mapping of the JCTF data and fit the distribution of causal variant(s) as a function of distance to the transcription start site (dTSS) of the corresponding gene (Supplementary Figure S1). Specifically, we binned dTSS into 200 non-overlapping windows (each window size = 10 kbp), let x be the mean dTSS in the bin, and y be the probability of PIP > 0.9 in the bin, and fitted a line in the log–log space, to approximate the probability of a variant being ‘causal’ as a function of the dTSS. Then for each simulated eGene, we calculated the probability of each variant being causal based on the dTSS, and selected the causal variant(s) as a random draw from the probability distribution without replacement.

- *Random (non-cis-genetic) effects*

To simulate the expression of a gene with genetic heritability ${h_g}^2$, we first drew the random effect $\widehat {{ \epsilon _i}}$ from a normal distribution $N( {0,\sqrt {1 - {h_g}^{2}} } )$ for each sample $i$. Due to finite sample size (*n* = 465), the observed $\widehat \epsilon$ in reality has a mean and variance different from 0 and $( {1 - {h_g}^2} )$. We further correct for this by $\epsilon _{i}\; = \;( {\widehat { \epsilon _{i}} - mean( {\widehat {\epsilon} } )} ) \cdot \frac{{\sqrt {1 - {h_g}^2} }}{{std( {\widehat \epsilon } )}}$, so that the random effect has the desired mean and variance in an exact manner, while still being drawn from a normal distribution.

- *Genetic effects*

We then assign the fixed genetic effect from the causal variant(s). We assume that when there are > 1 causal variants for a gene, their effect sizes in the standardized unit are the same (i.e. the unstandardized per-dosage effect sizes are larger for rarer variants). Specifically, let $X$ be the standardized genotype matrix corresponding to the causal variant(s) [i.e. $( {465\; \times k} )$ matrix, where $k$ is the number of causal variants on the gene, ranging from 1 to 5; $X$ is a vector in the case of one causal variant, without loss of generality], $\beta$ is the effect size of each causal variant (scaler, same for each variant) and $R = {X^t}X\;/\;465$ is the covariance matrix (LD matrix) of $X$, then ${\beta ^2} = {h_g}^2/ \sum _{i,j \epsilon cis} {R_{i,j}}$ is determined for each causal variant.

- *Total effects*

Finally, we construct the simulated ‘expressions’ $y = X \cdot \beta + \epsilon$ for each gene.

### Knockoff genotype construction

Taking the genotype matrix as an input, we followed the SNPknock vignette instruction to construct a knockoff genotype per chromosome. As recommended in Sesia *et al.* (13), we did not include imputed variants.

We set the parameters K = 20, num_iter = 20, defined the group based on non-overlapping a 1 Mb window split of the genome and ran the SNPKnock algorithm. We then performed diagnostics such as measuring the correlation between real and knockoff phenotypes, all suggesting that our knockoff genotype basically satisfies the desired conditions (Supplementary Figure S3).

### FDP estimate utilizing the knockoff genotype

We defined the FDP for (*cis-*) eGene discovery in the simulation in the following way. For each gene, we focused on three 1 Mb regions (i.e. groups) in the genome proximal to the gene TSS; the group containing the TSS, and the groups immediately upstream and downstream. We define this 3 Mb window as the *cis-*region for the gene. We then tested the association with the gene expression for each of the variants in the *cis-*region from the real genotype data as well as the knockoff using tensorQTL (17).

**Figure 2.**
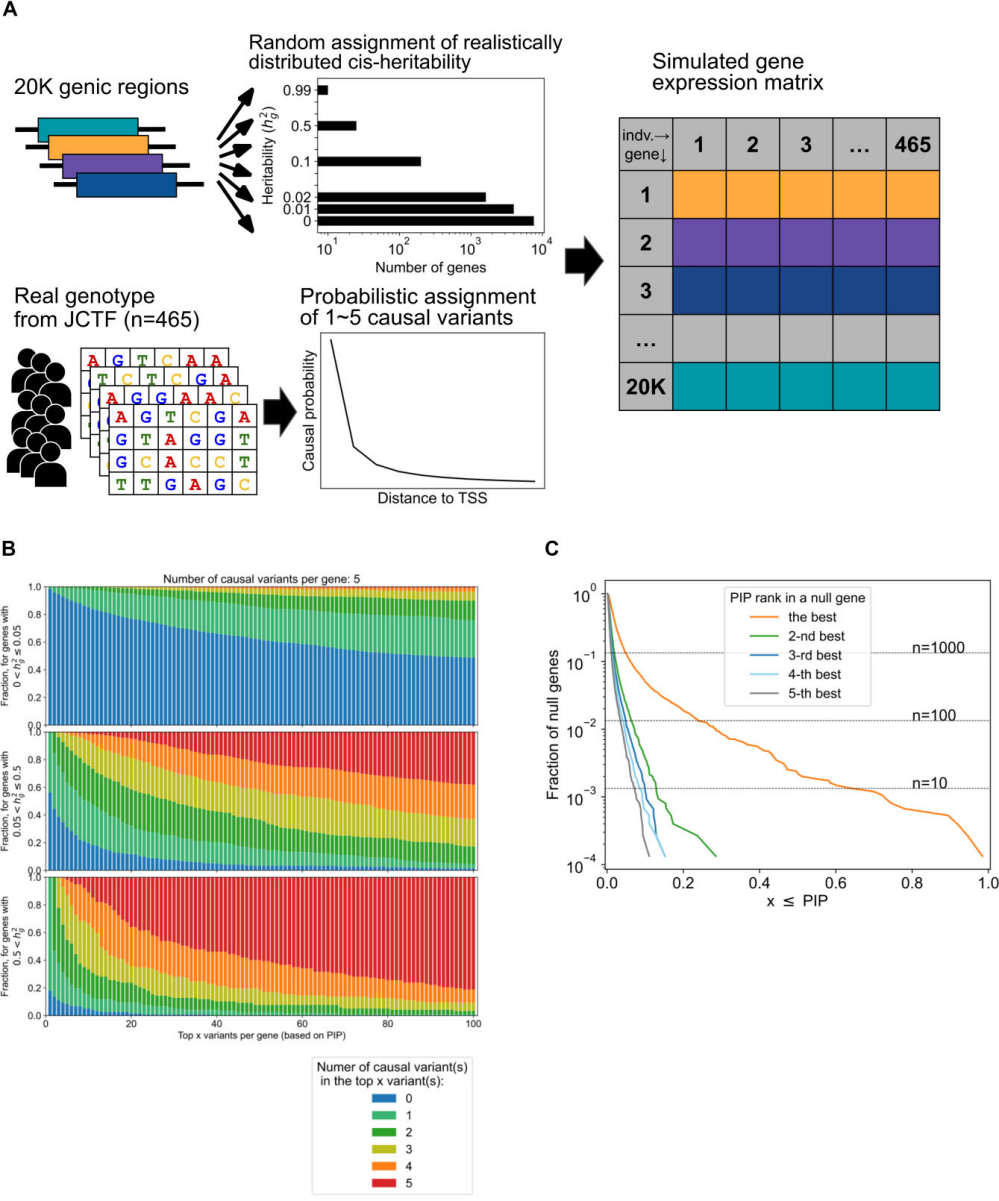
Simulating a gene expression matrix. (**A**) Overview of the simulation framework utilizing the real genotype data from the JCTF. Step 1, we randomly assign heritability to the genes matching a realistic distribution. Step 2, we randomly assign 1–5 causal variant(s) for genes with non-zero heritability, matching a realistic distribution as a function of the distance to the gene transcription start site (dTSS). Step 3, we simulate per-sample gene expression for each gene based on the heritability and causal configuration. (**B**) The number of prioritized causal variants in the top 10 PIP variants when five causal variants per gene are simulated, for low (top), middle (center) or high (bottom) heritability genes (red, all five causal variants are included in the top 10 PIP variants; orange: four out of five; yellow, three out of five; blue, zero out of five). (**C**) Distribution of PIPs for the top to the fifth best variants in null genes.

Next, we defined the test statistics ${W_g}$ for each gene as {$ - lo{g_{10}}( {min( {{p_{real}}} )} )\} \; - \;\{ { - lo{g_{10}}( {min( {{p_{ko}}} )} )} \}$. The test statistics, although different from the previous work (15,19) such as that utilizing a sparse regression model (14), match our desire in that (i) it would be sufficiently larger than 0 only if the window contains a real variant with low *P*-value, relative to that of knockoff variants in the window, and (ii) it would be symmetric around ${W_g} = 0$ under the null hypothesis. We note that our definition of a *cis-*region contains a larger set of variants than the typical definition of a ±1 Mb window from the TSS. We also note that combining test statistics across three groups violates the exchangeability assumption, which is guaranteed only for one group at a time. Nevertheless, we expect that extending the window size has minimal effect on the eQTL call as the existence of true causal variants right outside of the ±1 Mb window is a rare scenario. Our empirical results agree with this thought (e.g. Figure 2).

Finally, we defined the lFDP estimate as the function of test statistics $t$, which is the estimated number of negative ${W_g}$ divided by the positive ones around $| t |$, following the idea presented in previous work. Specifically, we sorted the positive ${W_g}$values, took a rolling window size of ±50 genes and defined the lFDP estimate given $t$ as;

$\frac{{number\;of\;genes\;where\; - ( {t + {\delta _1}} ) < {W_g} < - ( {t - {\delta _2}} )}}{{number\;of\;genes\;where\;\;( {t - {\delta _2}} ) < {W_g} < \;( {t + {\delta _1}} )}}$ , where ${\delta _1}$ and ${\delta _2}$ are chosen such that the denominator is exactly 100 and ${W_g}$ is the center.

For all the genes with ${W_g} \le 0$, lFDP was set to be 1. lFDP estimates that were larger than 1 were also set to be 1.

We note that our lFDP estimate will be exact only if there are no null genes with positive ${W_g}$ in the bin, which is an easy assumption for large enough ${W_g}$ but never true for small positive ${W_g}$. Our local lFDP estimate is thus in reality providing an upper bound instead of an exact point estimate. Also, the analysis of the real data from the JCTF was done in the same processes, except for the fact that covariates (60 PEER factors, 10 genotype PCs and sex) were included following the standard GTEx pipeline (https://github.com/broadinstitute/gtex-pipeline/tree/master/qtl) as well as our previous work of eQTL analysis in the JCTF (12). As shown in our previous work, the PEER factors significantly correlate with biological variations such as age or COVID-19 severity, and are thought to be effective in mitigating non-genetically determined variation of gene expression in eQTL discovery.

### Fine-mapping

FINEMAP with the default parameter setting and an in-sample LD matrix (adjusted for covariates when working with the real gene expression data in the JCTF) were used to perform fine-mapping of real and simulated data. The output PIPs from FINEMAP were defined as the ‘raw PIPs’.

The adjusted PIPs are calculated as follows:

Bonferroni: PIPs for any genes with minimum *P* > 5e-8 were set to be 0.
EMT: PIPs for any genes with minimum *P* > EMT threshold were set to be 0. The EMT threshold was defined for each gene as 0.0.5 divided by the number of independent tests estimated from the EMT algorithm (calculated using tensorQTL).
*q*-value: PIPs for any genes with *q*-value > 0.05 were set to be 0. Gene-level *q*-values were calculated using tensorQTL.
Knockoff: for each gene, knockoff-adjusted PIP = raw PIP multiplied by (1 – gene-level FDP), where gene-level FDPs were calculated using Knockoff.
Knockoff + Bonferroni: adjusted PIP = knockoff-adjusted PIP for any genes with minimum *P* > 5e-8, and is the raw PIP for genes with minimum *P* < 5e-8.

Fine-mapping with a non-uniform prior (without shrinkage) was performed as follows. For each gene, for the variants existing in GTEx, the PIP from GTEx calculated by FINEMAP was directly used as the prior. For the variants missing in GTEx, the prior was imputed with the mean of the non-missing values. After constructing the prior, FINEMAP was run with the *-prior-snps* flag to reflect the prior (i.e. let the causal configuration probability in the shotgun stochastic search of FINEMAP be dependent on the prior of the variants included in the configuration). For fine-mapping with a non-uniform prior with shrinkage, we first applied the same imputation process, and then any priors that are smaller than the maximum/100 (in a gene) were set to be maximum/100. We then normalized the priors so that the sum is equal to 1. The process was restricted to 6318 genes containing a variant with PIP > 0.01 in GTEx whole-blood data. When the KFc method was applied, the PIPs calculated from the above step were further adjusted by multiplying the (1 – FDP) for the corresponding gene in the JCTF.

### Simulating non-uniform priors

Using the simulation dataset constructed as in the Simulation setup section, we first selected 1025 eGenes with exactly one simulated causal variant harboring reasonably high (${h_g}^2$>0.05) heritability. We then additionally selected 512 null genes (randomly sampled from all the null gene pools, so that the number of genes in each of the three scenarios below will be roughly equal), and simulated three cases: (1) the true causal variant has the highest prior (for random *n* = 513 eGenes); (2) a non-causal variant on an eGene has the highest prior (for the rest *n* = 512 eGenes); and (3) a non-causal variant on a null gene has the highest prior (for the *n* = 512 null genes), with three different magnitudes of maximum prior (0.01, 0.1 or 0.9). Priors for other variants are randomly sampled from the PIP distribution in the simulation truncated at PIP < 0.01. After constructing the prior, FINEMAP was run with the -prior-snps flag to reflect the prior. When the KFc method was applied, the PIPs calculated from the above step were further adjusted by multiplying the (1 – FDP) for the corresponding gene in the JCTF.

### External data validation

The GTEx fine-mapping data from *n* = 670 whole-blood samples as well as other tissues were quantified as part of our previous study (5) (where KFc is not applied in the fine-mapping process). The EMS was calculated in our previous study (5), downloaded from https://www.finucanelab.org/data and was annotated using variant (hg38) and gene as the keys. The raQTL data were downloaded from https://sure.nki.nl. The Biobank Japan (BBJ) fine-mapping data we generated in our previous study (https://www.medrxiv.org/content/10.1101/2021.09.03.21262975v1) were downloaded from https://humandbs.biosciencedbc.jp/hum0197-v5-79. The study included 179 000 samples. Any variant presenting PIP > 0.1 in our standard fine-mapping pipeline (without KFc being applied) for any of the 13 hematopoietic traits (neutrophil count, mean cell hemoglobin, monocyte count, platelet count, lymphocyte count, red blood cell count, mean corpuscular hemoglobin concentration, eosinophil count, whole blood cell count, hematocrit, mean corpuscular volume, hemoglobin level and basophil count) were deemed as a BBJ ‘hit’. For the raQTL and BBJ data, since they were defined per variant, our eQTL fine-mapping data were first collapsed to per-variant (maximum) PIP, and were merged with the raQTL and BBJ data using hg19 as the key.

### Statistical analysis

All the statistical tests were two sided. No adjustment was made for the *P*-values we report. Error bars denote the standard error of the mean (SEM) unless noted otherwise.

## Results

### Constructing a simulated gene expression dataset

We first constructed a realistic *cis-*eQTL dataset (Figure 2A), using post-QC genotype data from the JCTF study (12). We then assigned a simulated expression of each gene in the following scheme (with more details in the Materials and methods): the heritability of *cis-*eQTLs ranged from 0 to 0.99 in a step window size of 0.01 (Supplementary Figure S1a), where most genes are in the low heritability window (38.1% = 7595 genes have ${h_g}^2 = 0$). The heritability distribution reflects prior studies suggesting a limited heritability of *cis-*eQTLs in most genes (16,19). In particular, our heritability distribution approximates that from a twin-based RNA expression study (19). For 12 318 genes with non-zero heritability (= ‘eGenes’), we assigned 1–5 causal variants, each having the same effect sizes for simplicity, with a realistic distribution regarding the dTSS (5,11,20) (Supplementary Figure S1b). All the other effects [e.g. trans-eQTL effects (21)] are modeled as normally distributed noise, and covariates are not included in the model, for simplicity.

We then performed genome-wide fine-mapping of the simulated eQTL data using FINEMAP to assign (raw) PIPs for each variant gene pair. The distribution of PIPs matched our intuition (Figure 2B; Supplementary Figure S2). For example, when the heritability is high (>0.5), the top PIP variant is truly a causal variant for the vast majority of the genes (82.1% for the case of five causal variants per gene; Figure 2B bottom left). In contrast, when the heritability is low (<0.05), even for eGenes, nearly half of the genes do not contain causal variant(s) in the top 100 PIP variants (48.8% for the case of five causal variants per gene; Figure 2B top left), highlighting the fundamental difficulty of fine-mapping in low-heritability cases. Importantly, we observed non-negligible PIPs for variants on null genes (Figure 2C); >10 genes (16 genes, 0.21%) each harbored a variant with PIP > 0.5, and >100 genes (141 genes, 1.88%) each harbored a variant with PIP > 0.2. By definition, the ideal PIP for variants in null genes should be exactly zero, motivating us to use a strategy to flexibly take the probability of ‘no causal variants in a gene’ (i.e. probability of false eGene discovery) into account.

### Constructing a knockoff genotype for FDP estimation

**Figure 3.**
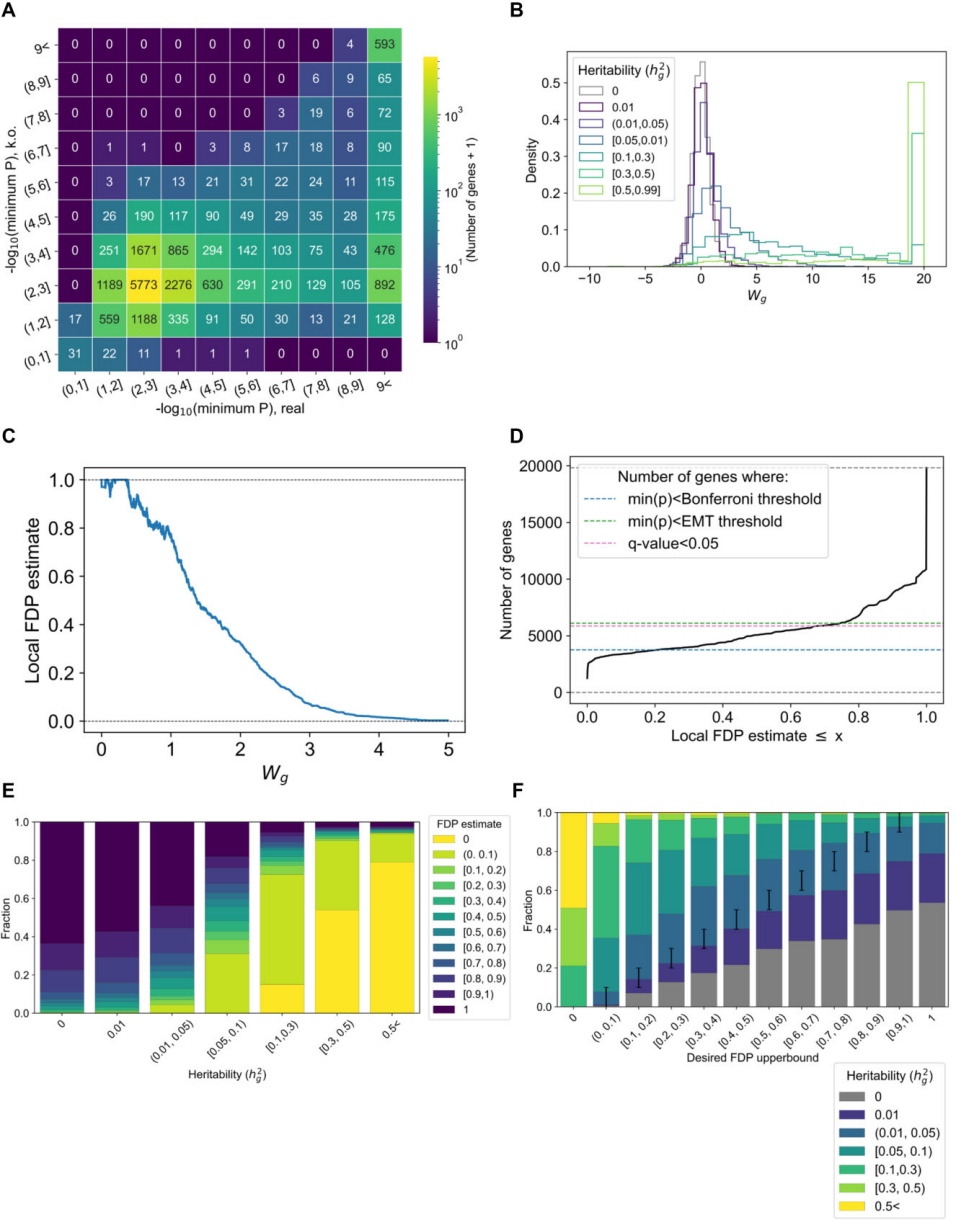
Knockoff allows eGene discovery with FDP calibration in simulation. (**A**) Heatmap comparing the –log10(minimum *P*-value) in the real genotype and corresponding knockoff genotypes. (**B**) Distribution of knockoff test statistics, reflecting the significance of the real genotype relative to the knockoffs, for different heritability bins. (**C**) Local FDP as a function of the test statistics. (**D**) Cumulative distribution of the number of genes as a function of the FDP threshold. The numbers of genes passing different significance thresholds are shown in dotted lines as a comparison. (**E**) Distribution of FDP for different heritability bins. (**F**) Distribution of heritability bins as a function of FDP bins.

To enable an estimation of FDP for each gene, we then constructed a knockoff genotype of the dataset, using the SNPknock package (13), with non-overlapping 1 Mb window resolution (Supplementary Fig. S3). To construct knockoff statistics for each gene, we performed an association test for each of the real variants in the *cis-*window, as well as the knockoff variants (Figure 3A; see the Materials and methods). Naturally, an eGene will probably have a low minimum *P*-value from the real genotype and a relatively large minimum *P*-value from the knockoff genotype. We thus take the differences of the minimum *P*-value between the real and knockoff genotype to define the knockoff test statistics per gene [${W_g}$; Figure 3B, C; we acknowledge that different methods exist for defining the test statistics; see Sesia *et al.* (14) and Supplementary Note]. As expected, the test statistics for ‘null’ genes (genes with *h*_g_^2^ = 0) are symmetric, whereas the distribution shifts towards ${W_g}$>>0 as ${h_g}^2$ becomes larger (Figure 3B; Supplementary Figure S3g, h). We calculated the fraction of genes with positive ${W_g}$ in a sliding window (Figure 3C), which provides us with a continuous distribution of lFDP estimates.

In our simulation, roughly half of the 19 913 genes (10 868 genes = 54.6%) had an lFDP < 1, and roughly a quarter (5069 = 25.5%) had an lFDP < 0.5, providing a larger set of genes compared with min(*P*)<5e-8 (Figure 3D).

### Knockoff enables calibration of lFDP for eGene identification

We evaluated the quality of our lFDP estimation in two ways. First, we examined the distribution of lFDP estimates per heritability (Figure 3E). A total of 96.2% of the null genes had an lFDP > 0.5 (=7269 out of 7556 null genes, with 4804 genes = 63.6% having an lFDP = 1; Figure 3E leftmost bar), and 95.9% of the high-heritability (${h_g}^2$>0.5) genes has an lFDP < 0.5 (= 720 out of 750 genes, with 600 genes = 79.9% having an lFDP of exactly 0), consistent with our desire to minimize the discovery of null genes while maximizing the discovery of non-null genes.

Second, to evaluate the calibration of our lFDP estimation, we examined the distribution of heritability for each lFDP bin (Figure 3F). The proportion of ‘false’ discovery (i.e. the proportion of null genes) was constantly lower than the lFDP bin lower bound, suggesting that our knockoff statistics successfully control the lFDP upper bound [while perfectly matching the lFDP is challenging in practice, as described in Sesia *et al.* (15)]. We also examined the distributions when applying hard-thresholding based on adjusted *P*-values (Supplementary Fig. S4), each having a different false and true discovery rate of eGenes, without a theoretical and practical guarantee of lFDP.

Our simulation overall suggested that knockoff controls the lFDP while successfully prioritizing large amounts of eGenes, especially when the heritability is high enough.

### The KFc method enables calibration of PIP for causal variant identification

**Figure 4.**
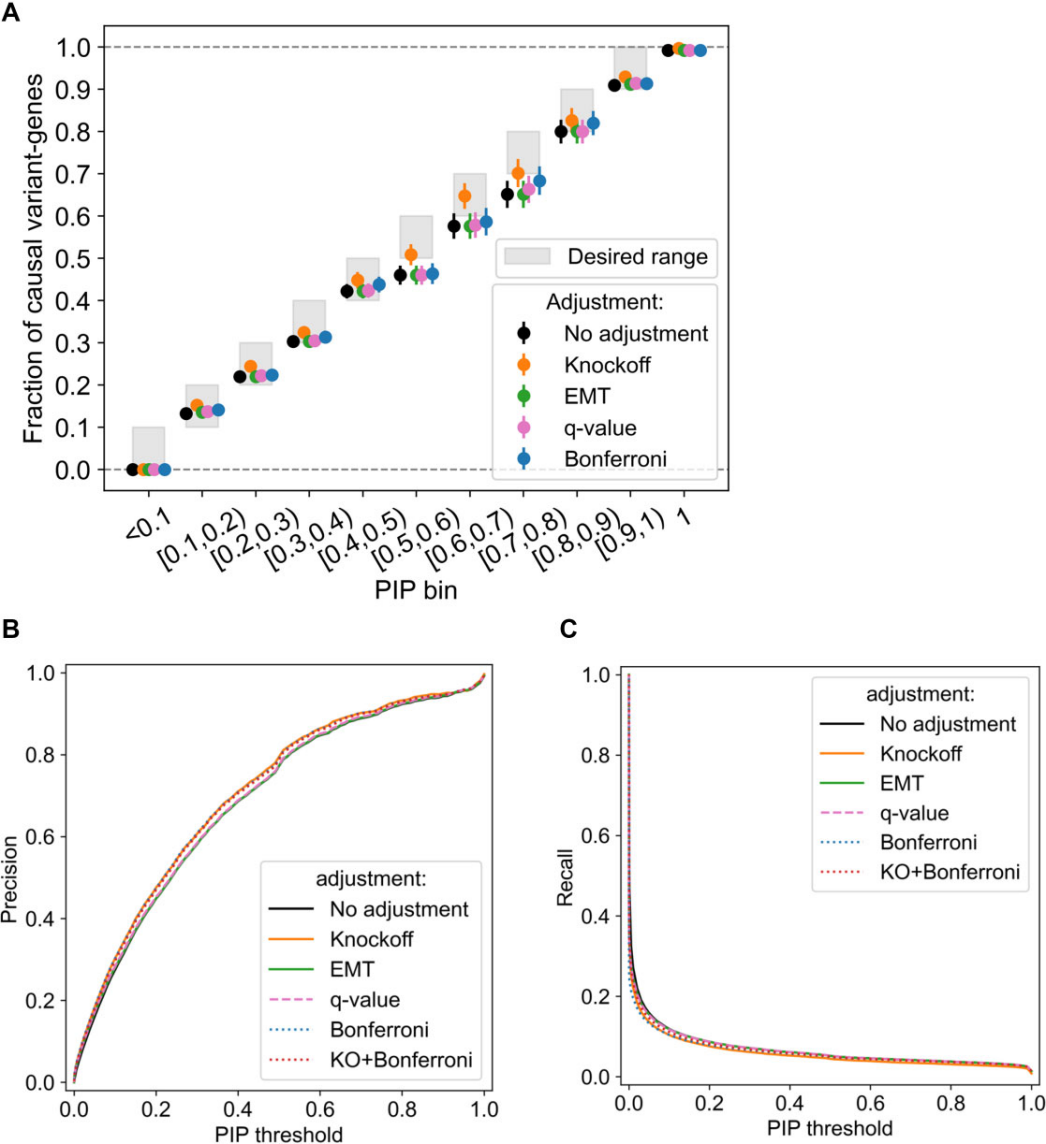
The KFc method assigns conservative and calibrated PIP in simulation. (**A**) Fraction of causal variant genes for different PIP bins, when using various PIP adjustment methods including the KFc. The desired fractions are shaded. (**B**) Precision of causal variant gene prioritization as a function of PIP threshold for different adjustment methods including the KFc. (**C**) Recall of causal variant gene prioritization as a function of PIP threshold for different adjustment methods including the KFc.

We next shifted our attention closer to the final task, which is to fine-map the causal variant(s) in eGenes. To this end, we investigated the fraction of true causal variants in different PIP bins, with or without different adjustment methods applied (Figure 4A; Supplementary Figure S5). KFc resulted in better calibration of PIP compared with other methods such as raw PIP, *q*-value (22,23) or Bonferroni-adjusted *P*-value based eGene discovery followed by fine-mapping (Figure 4A; KFc was the only method where the probability of true causal variants was within the lower and upper bound across different PIP bins).

When we compared the precision and recall of causal variant discovery as a function of PIPs (Figure 4B, C; Supplementary Figure S6), KFc achieved the highest precision (area under the PIP–precision curve = AUPPC = 0.7047; Figure 4B), slightly higher than Bonferroni correction (the second best, AUPPC = 0.7038) as well as the other correction methods such as EMT (24) or *q*-value-based methods (AUPPC < 0.6910). When testing across different hyperparameter and random seed settings, KFc consistently presented precision comparable with the Bonferroni method and higher than the others (0.7025 < AUPPC and 0.7039 on average; Supplementary Figure S6i). These multiple knockoffs also allow us to further improve the precision by smoothing individually noisy FDP estimates, as suggested in (18) (AUPPC = 0.7074; Supplementary Figure S6j). We note that the high precision was achieved with a sacrifice of recall (area under the PIP–recall curve = 0.6587; Figure 4C; Supplementary Figure S6k, l), where the KFc was the least powered method. We assume thatr this is due to incompleteness of knockoff genotype construction, which by chance assigns a very high FDP estimate to an eGene with an extremely low *P*-value. Indeed, combining KFc with Bonferroni correction resulted in a significantly better performance in terms of both precision and recall (red line in Figure 4B and C).

Our simulation overall showed that KFc allows better calibration of PIP, and allows us to prioritize putative causal variants with a precision comparable with the stringent Bonferroni correction and higher than alternative methods when applying the same PIP threshold (with a certain loss of recall, presumably due to incompleteness of knockoff genotype generation; Supplementay Figure S7; Supplementary Note).

### The KFc method applied to real data possibly improves fine-mapping precision

**Figure 5.**
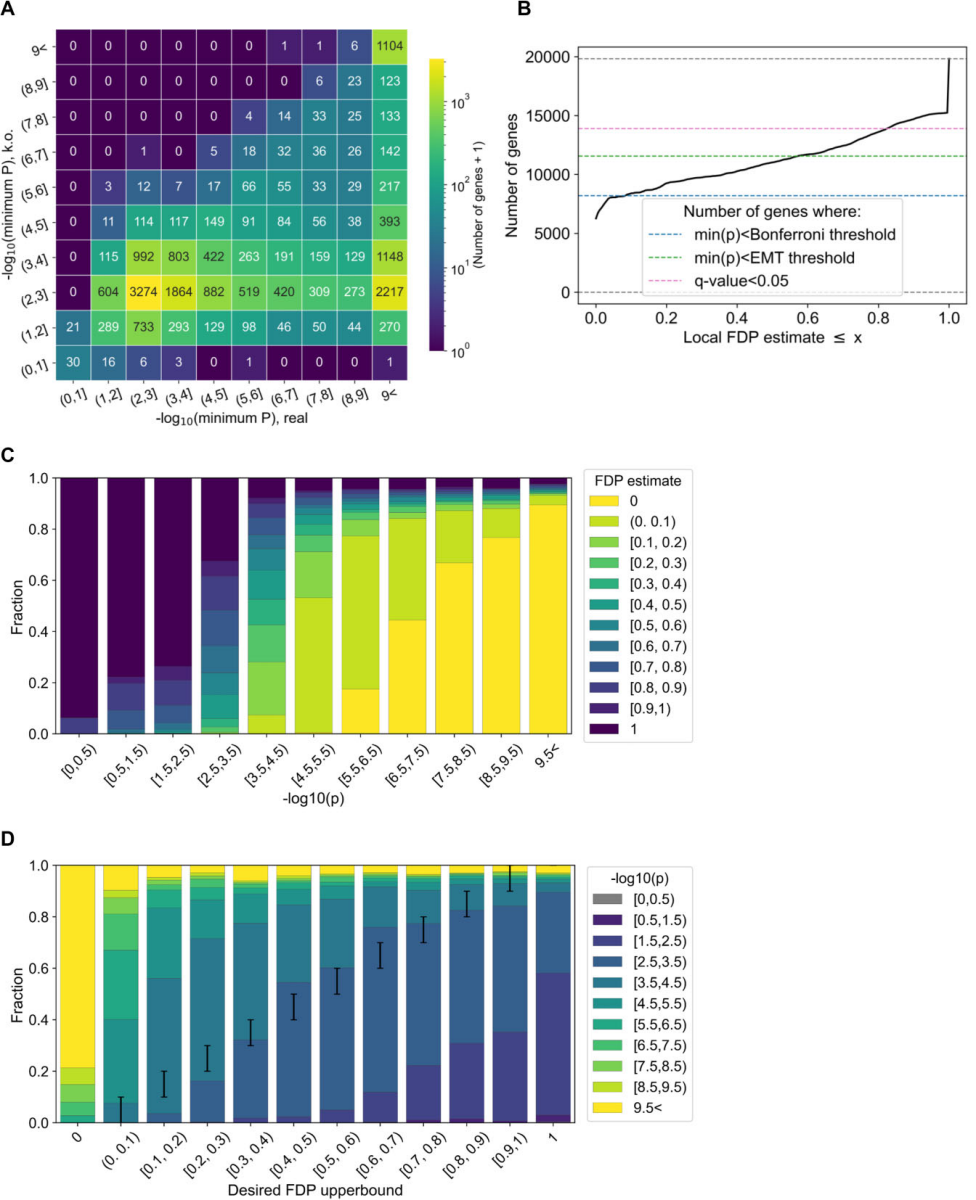
Overview of the KFc method applied to gene expression data from the JCTF. (**A**) Heatmap comparing the –log10(minimum *P*-value) in the real genotype and corresponding knockoff genotypes, where the phenotype is the gene expression data from the JCTF study. (**B**) Cumulative distribution of the number of genes as a function of FDP threshold. The numbers of genes passing different significance thresholds are shown in dotted lines as a comparison. (**C**) Distribution of FDP for different minimum *P*-value bins. (**D**) Distribution of minimum *P*-values as a function of FDP bins.

Having evaluated the performance of our KFc approach, we next applied the method to the real gene expression data in the JCTF (Figure 5A; Supplementary Figure S8). The lFDP estimates were overall higher than that in the simulation, presumably thanks to the technical improvements in the RNA-seq data generation and QC compared with previous years (25) (we note that an exact match between the distribution of heritability in real and simulated data is not a requirement); >40% of the genes (8351 out of 19 913 = 41.9%) had an lFDP < 0.1 (Figure 5B). The significance of genes overall agrees with that of min(*P*), providing a gradation of lFDP along with the nominal *P*-value (Figure 5C). Most of the genes with lFDP = 0 have very low *P*-values (5818 out of 6265 genes = 92.9% are below 5e-8; Figure 5D), as expected. The KFc method uses these lFDP estimates to adjust the raw PIPs from the FINEMAP algorithm with uniform prior.

**Figure 6.**
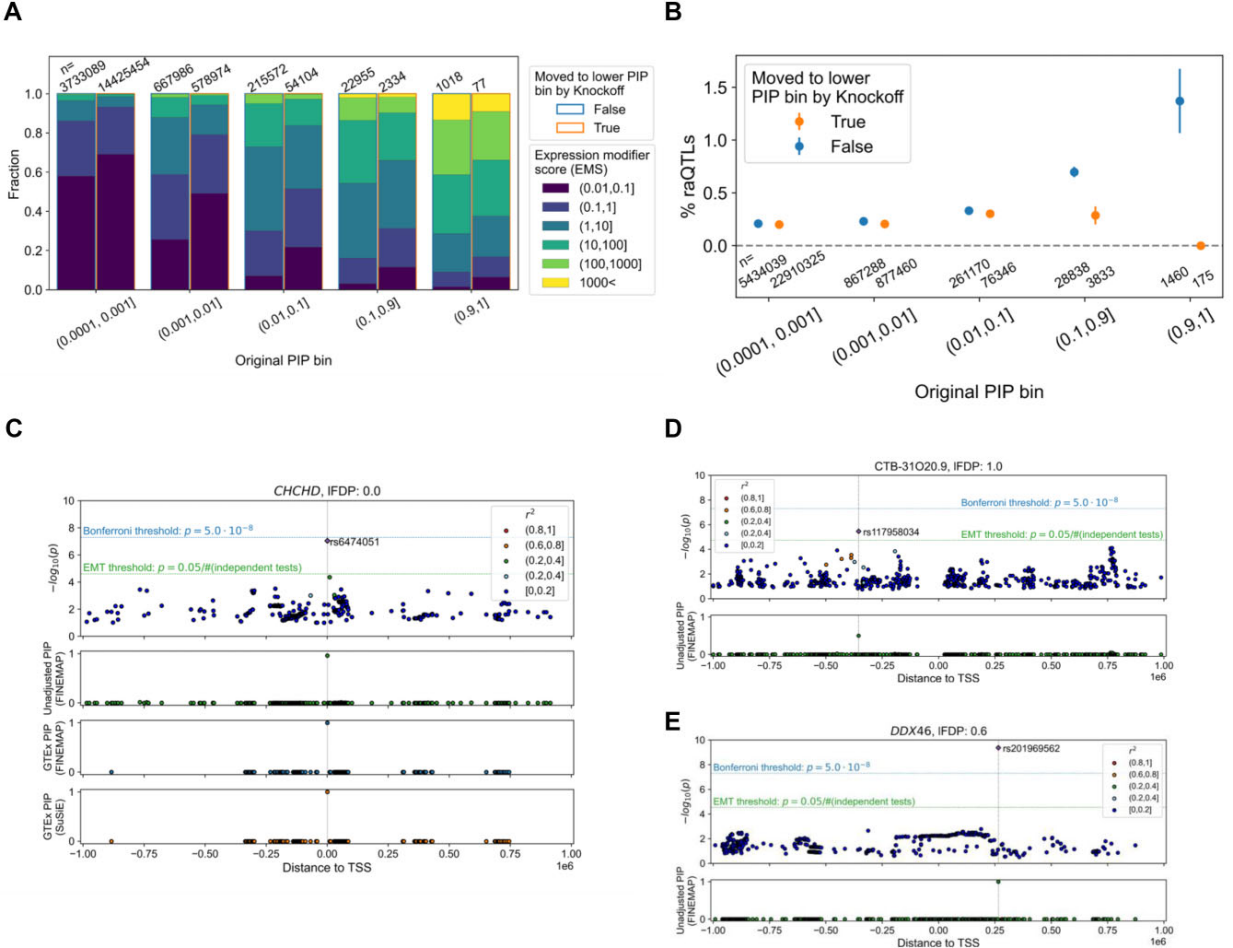
Biological characterization of the KFc method applied to gene expression data from the JCTF. (**A**) Distribution of the EMS, for different PIP bins, comparing the variant genes where the KFc method resulted in significantly lower PIPs (orange edge = right side) versus those that did not (blue edge = left side). EMS reflects the functional evidence of a variant being causal to a gene. (**B**) Percentage of raQTLs for different PIP bins, comparing the variants where the KFc method resulted in significantly lower PIPs (orange dots) versus those that did not (blue dots). (**C**) –log10(*P*-value) and PIP before adjusting by KFc, as well as the PIPs from GTEx assigned by two different algorithms (FINEMAP/SuSiE) for *cis-*eQTL effects on the *CHCHD* gene. (**D** and **E**) –log10(*P*-value) and PIP before adjusting by KFc for *cis-*eQTL effects on the *CTB-31O20.9* and *DDX46* gene. Local FDP estimates for each gene are shown at the top.

We evaluated the KFc method-derived adjusted PIP based on two near orthogonal functional genomics-based scores. First, we examined the distribution of the EMS (5). EMS is trained on putative causal variants (PIP in FINEMAP and SuSiE > 0.9) of Bonferroni-significant eGenes [min(*P*)<5e-8] in GTEx utilizing > 5000 functional features, such as dTSS and deep neural network-derived features (26), and thereby works as a proxy for variant-level functional evidence of causality. When we compared the unadjusted versus adjusted PIP, 77 variant genes with unadjusted PIP > 0.9 resulted in adjusted PIP < 0.9. Compared with the 1018 variant genes that remained at PIP > 0.9, those 77 variants seemed to have a higher probability of being in the smallest EMS bin and a lower probability of being in the largest EMS bin (Figure 6A, rightmost bars). Such an observation, suggesting that the KFc method successfully assigns smaller PIPs to a fraction of probably false causal variants, was consistent for all the other PIP bins (χ^2^ test *P* < 1e-100 collectively, excluding those already in the lowest PIP < 0.0001 bin for fair comparison; Figure 6A).

Second, when we compared the proportion of functional variants validated in a reporter assay [reporter assay eQTLs = raQTLs (15)], variants shifted to lower PIP bins constantly showed lower probability of being raQTLs compared with those which remained in the same PIP bins (Figure 6B). For example, >1.4% of the 1327 variants that constantly remained in the highest PIP bin were raQTLs, whereas none of the 154 variants moved to lower bins were raQTLs (*P* = 3.20e-4 for Fisher's exact test combining the top two bins; such a difference was not observed when shuffling the FDP assignment; Supplementary Figure S9a). Moreover, these depletions of EMS and raQTL hits were consistent even when we stratified the genes by their minimum *P*-value (whether *P* < 5e-8 or not; Supplementary Figure S9b–e), highlighting the utility of KFc over simple hard-thresholding based on the *P*-value.

The results altogether suggest that the KFc method is likely to be utilized to improve the accuracy of eQTL fine-mapping in real data.

### Examples of causal variant discovery based on the KFc method

Here we show three examples of genes that seem to benefit from the KFc method compared with canonical thresholding approaches.

The first example suggests missed opportunities when using the most strict Bonferroni threshold. rs6474051 on gene *CHCHD* (Figure 6C) has a –log10(*P*-value) slightly less than the Bonferroni threshold (9.1e-8 > 5.0e-8 and therefore not defined as an eGene in a stringent filtering). Since the lFDP estimate for this gene is 0, we can apply FINEMAP and the PIP is near one (0.96). rs6474051 is also putatively causal in GTEx (PIP > 0.999 in both SuSiE and FINEMAP), increasing the support that it is a true causal variant.

The second example suggests a possible false positive when using a looser threshold (such as EMT-adjusted *P* < 0.05). The lead variant rs117958034 for transcript *CTB-31O20.9* has a –log10(*P*-value) passing the EMT threshold (3.4e-6 < 1.8e-5 = 0.05/2747; Figure 6D). However, this variant is extremely rare [missing in a non-East Asian population and observed only at minor allele frequency = 0.0065 in gnomAD (27)], with a peak in the read depth < 15 specific to carriers, suggesting a possible sequencing error. Also considering the fact that the variant is 354 171 bp upstream of the transcript TSS, we assume that there is a low chance that the variant is a causal variant. Matching our intuition, the KFc method assigned PIP = 0 since the lFDP for this gene is 1.

The third example, rs201969562 for the gene *DDX46*, is a nuanced one (Figure 6E). The variant has –log10(*P*-value) passing the threshold and is identified as a putative causal variant even with the most stringent traditional threshold (Bonferroni). However, this is a rare variant without previously known major disease or phenotype associations, and carriers are also trending towards low depth in gnomAD, suggesting a possible sequencing error. Our knockoff-based adjustment decreases the PIP by roughly half (based on lFDP = 0.6), avoiding deterministic PIP ∼1 or ∼0 (raw PIP = 1, KFc-adjusted PIP = 0.4). GTEx fine-mapping did not suggest any putative causal variant for the gene.

These examples highlight cases where the KFc method allows rescue of putative causal variants not passing the Bonferroni threshold, avoids false positives passing a looser EMT threshold, or assigns medium-high PIP for variants with medium confidence, instead of deterministic PIP = 1 (when using EMT) or 0 (when using Bonferroni), all highlighting the utility of the KFc method compared with other alternatives using thresholding approaches.

### Simulating a non-uniform prior provides insights into cross-population fine-mapping

We next considered utilizing the KFc method when using a non-uniform prior, considering the context of cross-population fine-mapping. In particular, we considered a situation where fine-mapping results from an external study of a different population background are publicly available (another common scenario, which is outside of the focus of this work, is functionally informed fine-mapping). In such a situation, one simple integration method often used is to set the PIPs from the external study as a prior when performing fine-mapping of the study population. Huffman *et al.* (28) used PIPs from a study based on European samples (29) as a prior to perform statistical fine-mapping of the association statistics from another study of African origin. While in their case the two-step method allowed them to increase the power of fine-mapping and suggest a common putative causal variant possibly responsible for both the *OAS1* splicing pattern and COVID-19 severity, in reality we could also imagine a situation where too strong priors from one study result in inflation of PIP in another study (even in their case, uncertainty exists in that the putative causal variant is not the lead variant in the African dataset). We hypothesized that conservative estimation using KFc would be beneficial in such contexts.

**Figure 7.**
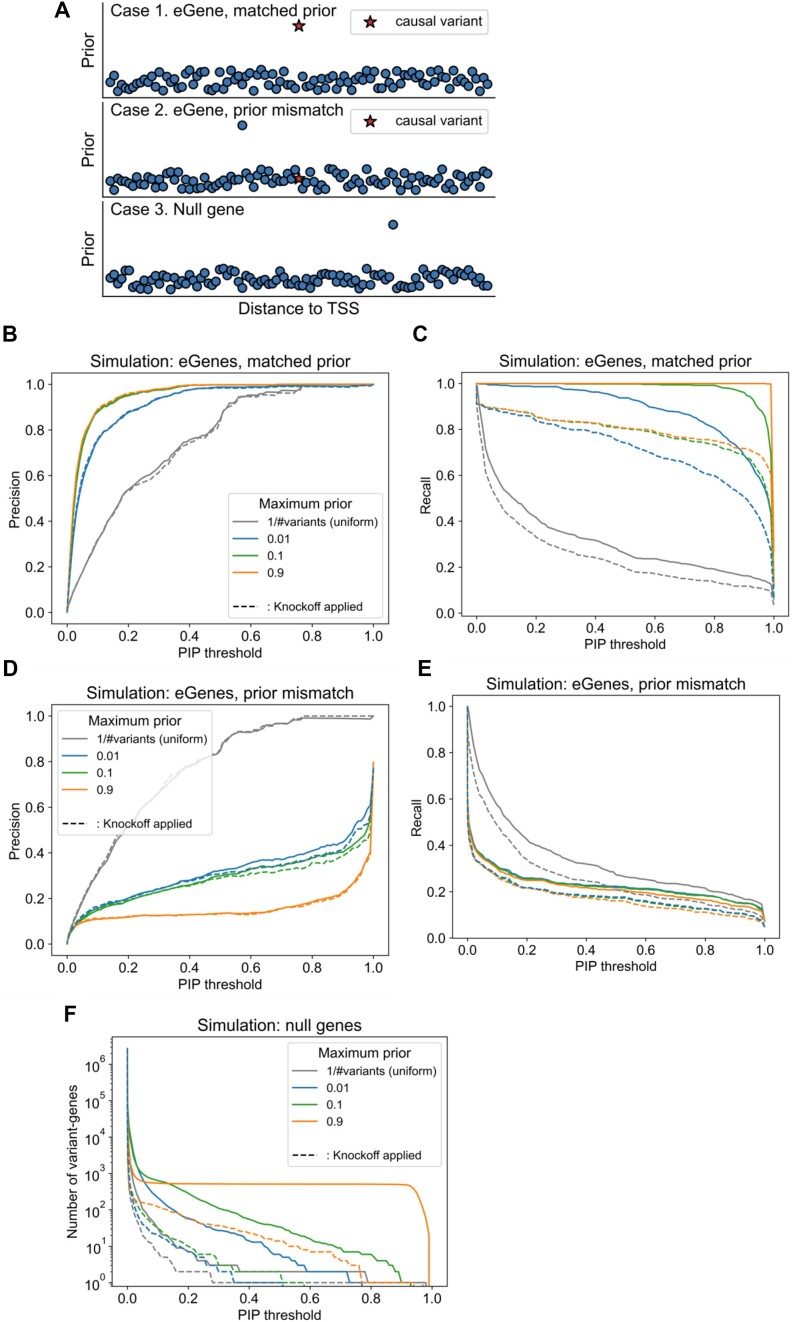
Performance of KFc when using a non-uniform prior of different strength in simulations. (**A**) Overview of the simulation, where we considered three scenarios (case 1: the causal variant has the strongest prior in an eGene; case 2: a non-causal variant has the strongest prior in an eGene; and case 3: a non-causal variant has the strongest prior in a null gene with no heritability) and three strengths of the prior (max = 0.01, 0.1 and 0.9). (B and C) Precision (**B**) and recall (**C**) as a function of PIP, with or without the KFc method applied, for scenario 1. (**D** and **E**) The same for scenario 2. (**F**) Number of variant genes above the PIP threshold as a function of PIP, for scenario 3, where the genes are null genes and thus the ideal PIP should be 0 for all the variant genes.

To test the hypothesis, we simulated different priors and tested the performance of fine-mapping under canonical versus the KFc method. In brief, we simulated three cases: (1) the true causal variant has the highest prior; (2) a non-causal variant on an eGene has the highest prior; and (3) a non-causal variant on a null gene has the highest prior, with varying magnitude of maximum prior (0.01,0.1 or 0.9; see the Supplementary Methods for details). The results were overall consistent with our intuition. In the first scenario (eGenes, matched prior), a higher prior on the causal variant resulted in higher precision and recall (Figure 7B, C). For example, when the prior was 0.9, the precision was exactly 1 up to a threshold of PIP = 0.69, and a recall rate 0.998 was achieved at a stringent threshold of PIP = 0.99. We note that the performance decrease was mild even when the prior was 0.1 (again achieving 100% precision up to PIP = 0.69, and a recall rate 0.8 at PIP = 0.98). In the second scenario (eGenes, prior mismatch; Figure 7D, E), on the other hand, a higher maximum prior, which is on a non-causal variant, severely decreased the fine-mapping performance. For example, the precision and recall were as low as 0.23 and 0.14 at the PIP = 0.9 threshold, when the maximum prior was 0.9. Importantly, applying the KFc method (i.e. post-hoc adjustment of the PIPs based on FDP estimate) resulted in a slight decrease of performance, possibly due to accidentally assigning high FDP to a subset of eGenes. Our simulation nevertheless highlights the value of the KFc approach, as it significantly reduces the number of variant genes with high PIPs in the case of null genes over a range of maximum prior values (Figure 7F). For example, when the maximum prior was 0.9, a total of 494 variant genes (i.e. most of the top prior variants for 512 genes) had a PIP >0.9, even though they are non-causal. Applying KFc reduces the number from 494 to only 1, by taking into account the fact that those genes, even though they have a variant with a high prior, are most likely to be null genes (characterized by a high FDP).

When utilizing fine-mapping results from an external study as a prior, we do not know the probability that the causal variants are biologically consistent and exist in both populations with detectable effect sizes. In other words, we do not know which of the three scenarios above are the most realistic and dominant. Nevertheless, assuming that there is a non-negligible chance that the variant with the highest PIP in an external study is not the causal variant in another study, our simulation highlights the value of KFc to reduce the number of false discoveries, and also warns us that directly using PIPs from external studies without shrinkage could increase false discoveries.

### Application of KFc in cross-population fine-mapping

Finally, we performed cross-population fine-mapping utilizing the KFc method in real data, targeting 6318 genes containing a variant with PIP > 0.01 in GTEx whole-blood data—a dataset derived from roughly the same sample size and identical tissue (Supplementary Figure S10: Supplementary Methods).

**Figure 8.**
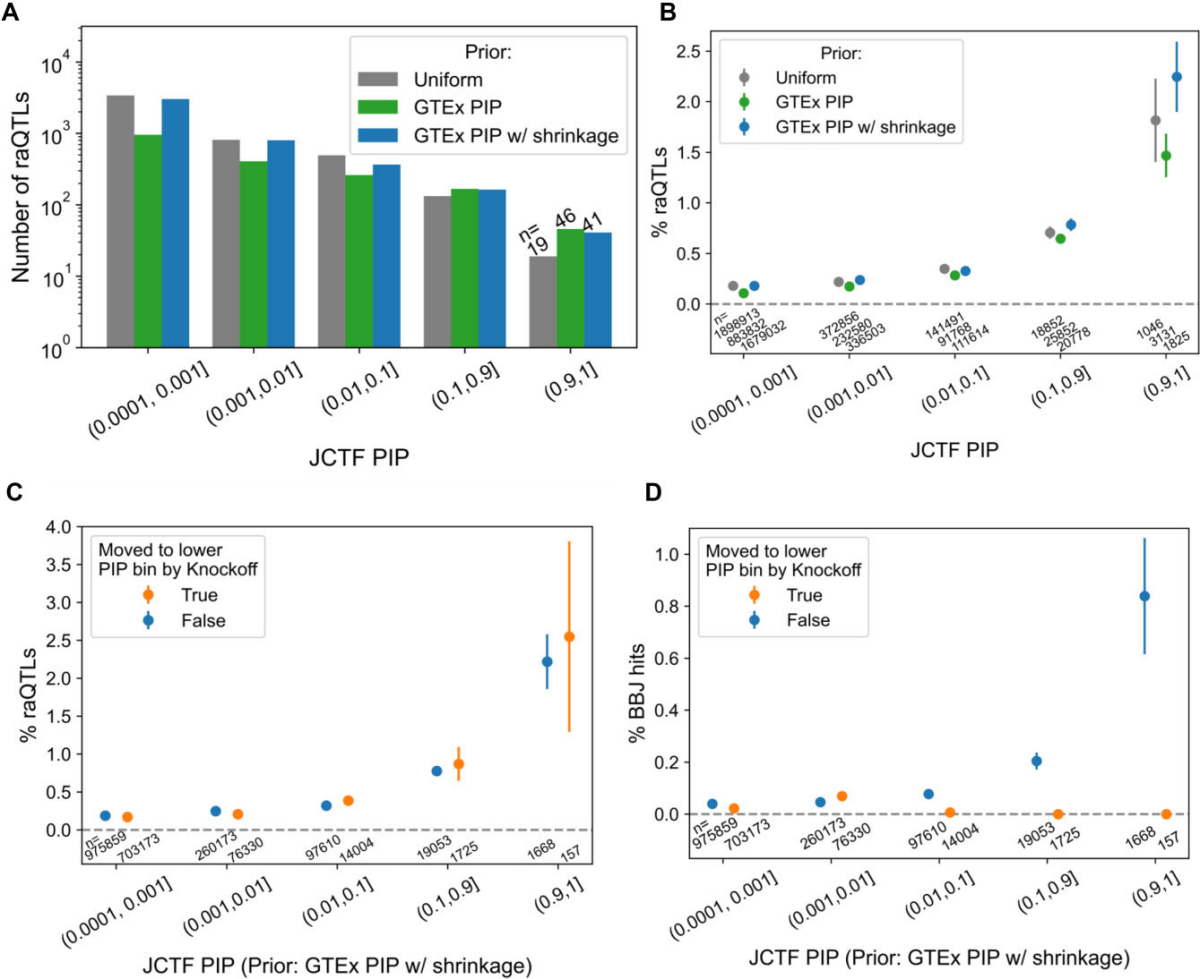
Utilizing an external fine-mapping dataset as a prior in combination with the KFc method. (**A**) Number of raQTLs for different PIP bins, when using a uniform prior (gray, left bar), or GTEx PIP as a prior without shrinkage (green, center bar) or with shrinkage (blue, right bar). (**B**) Percentage of raQTLs for different PIP bins, when using a uniform prior (gray dots), or GTEx PIP as a pror without shrinkage (green dots) or with shrinkage (blue dots). (**C**) Percentage of raQTLs for different PIP bins, comparing the variants where the KFc method resulted in significantly lower PIPs (orange dots) versus those that did not (blue dots). (**D**) Percentage of possible causal variants (PIP > 0.1) for hematopoietic traits in the BBJ for different PIP bins, comparing the variants where the KFc method resulted in significantly lower PIPs (orange dots) versus those that did not (blue dots). In (C) and (D), the PIPs were calculated using GTEx PIP as a prior with shrinkage, and further adjusted with knockoff-derived FDP when the KFc method was applied.

We first compared the performance of fine-mapping of JCTF data with a uniform prior versus GTEx PIP as a prior (with and without shrinkage). Enrichment of raQTLs was used as the evaluation, since EMS is not available for the variants missing in GTEx, which introduces significant biases if used. Using GTEx PIP as a prior resulted in a larger number of raQTLs in the high PIP bins, in the case of both with and without shrinkage (e.g. from 19 to 43 or 47 raQTLs in the PIP > 0.9 bin; Figure 8A). The enrichment level was significantly higher when shrinkage was applied (*P* = 0.037 for the top two bins combined, Fisher's exact test; Figure 8B). These observations suggested that cross-population fine-mapping, when priors are shrunk properly [as in Weissbrod *et al.* (30)], allowed discovery of a larger number of likely true causal variants, probably without loss of precision.

We then tested whether post-hoc PIP adjustment with the KFc method further improves the fine-mapping quality. When comparing the raQTL enrichment level, neither the top nor the second top bin showed significant depletion. We assumed that this is due to an ascertainment bias such that variants tested for raQTLs are enriched for common variants in European populations, and thus, as an additional benchmark, we also tested the enrichment level of possibly causal variants for hematopoietic traits (PIP > 0.1) in the BBJ (31). We observed depletion of such BBJ hits for variants moved to lower PIP bins for all the top three bins (down until PIP > 0.01, Fisher's exact test *P* = 2.15e-6 collectively; Figure 8D) as well as those moved to below the smallest PIP threshold (0.0001) (*P* = 7.16e-10). These observations suggest that KFc may be utilized to improve the precision of fine-mapping in the context of cross-population fine-mapping as well, although further inspection in addition to method improvements are warranted.

## Discussion

Fine-mapping of *cis*-eQTLs typically focuses on ‘eGenes’ (genes with likely true eQTL signals). Defining an eGene based on test statistics is non-trivial and, moreover, binary thresholding of test statistics to define eGenes poses a discontinuity that does not reflect human biology. In addition, the trade-off between discovery power and precision in fine-mapping when including all the genes versus focusing on eGenes has not been explored in depth.

In this work, we utilized the knockoff-based FDP estimation method to assign a continuous spectrum of eGene confidence, and further proposed a method combining it with fine-mapping algorithms, by utilizing the FDP estimate as a prior (Knockoff-Finemap combination = KFc). We constructed a realistically simulated genotype–gene expression dataset of nearly 500 samples (*n* = 465) to evaluate the validity of FDP estimation as well as the performance of the KFc method compared with other alternatives based on binary thresholding. We also showed that KFc improves the quality of fine-mapping in a real data example by investigating orthogonal functional annotation enrichments. We then extended our simulation and real data analysis to the context of cross-population fine-mapping, confirming the benefit of shrinking the priors consistent with previous work, and again suggesting the utility of KFc.

Our work, although presenting a refinement of existing fine-mapping strategies, has limitations. First is the quality of the knockoff genotype. Although we showed that the exchangeability measured by the LD between the real and knockoff genotype roughly holds true, the discrepancy between the LD is observed, especially when the allele frequencies are low (Supplementary Figure S3). Combined with the fact that we did not include rare (minor allele frequency < 0.01) and/or imputed genotypes in the knockoff construction, we assume the power loss of our KFc method in simulation can be partially attributed to the quality of the knockoff genotype (Supplementary Figure S7). Second, we note that the local FDP estimation utilizing the symmetry of test statistics under the null hypothesis, although it intuitively works, is still under development according to the original authors (14). Third is the simplicity of our simulation framework, which is not likely to be fully capturing the complex nature of genetic effects. Finally, the cross-population fine-mapping method that we tested utilizing PIPs from an external study as a prior is still naive (e.g. choice of the level of shrinkage). Future work should address these points to further improve the value of our proposed method, which includes the possibility of using methods other than knockoff for gene-level FDP estimation (32,33).

We envisage the following next steps also to be valuable. (i) Extension to complex trait causal variants. Defining a locus to fine-map for complex trait study based on the *P*-value and LD of variants in a region is fundamentally difficult, where we assume acquiring test statistics based on knockoff could be utilized, as done in (18). (ii) Extension to *trans*-eQTLs is trivial, which we are working on as another project. (iii) Incorporating functional annotation as done before (6,31), possibly as a prior to improve the power in both eGene discovery and fine-mapping would be meaningful (for more discussion on limitation and future work, see the Supplementary Note).

Finally, we note that our method in a way is a compromise between a frequentist approach (knockoff) and a Bayesian approach (downstream fine-mapping). The developers of the knockoff method further proposed to use it to filter down to single variant resolution for the purpose of fine-mapping (i.e. ‘non-Bayesian’ fine-mapping utilizing knockoff), while this manuscript focuses on improvement of Bayesian fine-mapping with the help of frequentist approaches. Direct comparison of purely knockoff-based fine-mapping versus our method, although very exciting, is outside the scope of our manuscript.

Along with the increase in the number and the scale of complex phenotypes and various molecular QTL studies, the importance of distinguishing between truly functional causal variants and tagged non-causal variants with zero or minimum functional effects via statistical fine-mapping is pronounced. Our work, improving the quality of a major fine-mapping algorithm by explicitly considering the probability of ‘no causal variant in a region’, serves as an important step forward in our effort to prioritize causal variants.

## Supplementary Material

lqad090_supplemental_files

## Data Availability

The RNA-seq expression matrix analyzed in this study is available at the National Bioscience Database Center (NBDC) Human Database (accession code: hum0343). The individual genotype data are available at the European Genome-Phenome Archive (EGA) (accession code: EGAS00001006284). The code used in the analysis is provided in github https://github.com/QingboWang/KFc (https://doi.org/10.5281/zenodo.8365949).
