# Supplementary material for: Estimating gene-level false discovery probability improves eQTL statistical fine-mapping precision
Source: NAR Genom Bioinform. 2023 Oct 30;5(4):lqad090. doi: 10.1093/nargab/lqad090 (PMC10616627; doi:10.1093/nargab/lqad090)
Supplement: lqad090_supplemental_files [file lqad090_supplemental_files.zip › NARGAB-2023-047_revised_supplementary.pdf]

Estimating gene-level false discovery probability improves eQTL statistical fine-mapping precision

Wang et al. 2023

Supplementary Information

Supplementary Note: pages 2 to 5
Supplementary Figures: pages 7 to 19
Supplementary References: page 20

Supplementary Note

Definition of false discovery rate and probability

Let $g_1, g_2, \ldots, g_N$ be N genes to fine-map, $t_1, t_2, \ldots, t_N$ be the corresponding significance metric (the larger the more significant), and $e_1, e_2, \ldots, e_N \in \{0,1\}$ be indicators of existence of causal variant(s) in each gene (1 if the gene is eGene, and 0 if it is a null gene). The local false discovery probability (lFDP) around a significance value t is defined as:
$lFDP(t) = 1 - \frac{\sum_{i \in T} e_i}{|T|}$, where $T = \{i \mid t - \delta \leq t_i \leq t + \delta\}$ is the set of indices for genes whose significance metric (nearly) equals t. In the ideal case where we have infinite numbers of observations, $\delta \to 0$. In practice we evaluated the calibration of lFDP by binning the lFDP estimation (e.g. **Fig. 3f**).

We note that this is different from the commonly used false-discovery probability (FDP), where we define T corresponding to genes whose significance metric is **equal or larger than** t:
$T = \{i \mid t \leq t_i\}$, as well as the false-discovery rate (FDR, which is the expectation of FDP).

Throughout the manuscript, we use the word lFDP to denote the local false-discovery probability. Alternatively, when we refer to a specific gene, we use the word gene level FDP without explicitly specifying the fact it is a local estimation.

Regarding various assumptions in major fine-mapping tools

Here we review the relevance of our proposed method to major fine-mapping algorithms.

FINEMAP: FINEMAP[1] assumes >0 causal variants in their model (We note that although the software allows the parameter `--prior-k0` corresponding to the null gene probability, the parameter is "*Only used when computing posterior probabilities for the number of causal SNPs but not during fine-mapping itself* " according to their documentation). We thus deemed FINEMAP as the algorithm where the KFc framework is most applicable, and used FINEMAP throughout our analysis.

SuSiE: SuSiE[2] also assumes >0 causal variants by default in their model, but does allow user-specified input of `null_weight` corresponding to the null gene probability. When `null_weight` is specified, each of the single effect vectors in the SuSiE model is multiplied by the null weight. Below we show that this is slightly conservative but approximately equivalent to the KFc scheme, where we adjust raw PIP output by multiplying by the null weight (i.e. the lFDP we estimate in KFc) post-hoc as shown below:

Let α_1 to α_L be L single effect vectors (e.g. typically we set $L = 10$) output from SuSiE algorithm and the PIP output from SuSiE model without null_weight be PIP_{raw}, according to the model,
$PIP_{raw} = 1 - \prod_{l=1}^{L}(1 - \alpha_l)$.

Let the estimated `null_weight` be k_0 , applying the KFc model (without re-running the SuSiE model with `null_weight` option) is equivalent to assigning $PIP_{KFc} = PIP_{raw} \cdot (1 - k_0)$.
Plugging k_0 as `null_weight` and re-running SuSiE will output:
$PIP_{adj} = 1 - \prod_{l=1}^{L}\{1 - (1 - k_0) \cdot \alpha_l\}$.
Sorting PIP_{raw} by the order of alphas,
$PIP_{raw} = 1 - (1 - \sum_{l=1}^{L} \alpha_l + \sum_{l1 \neq l2} \alpha_{l1}\alpha_{l2} - \sum_{l1 \neq l2 \neq l3} \alpha_{l1}\alpha_{l2}\alpha_{l3} + \ldots)$
$= (\sum_{l=1}^{L} \alpha_l - \sum_{l1 \neq l2} \alpha_{l1}\alpha_{l2} + \sum_{l1 \neq l2 \neq l3} \alpha_{l1}\alpha_{l2}\alpha_{l3} - \ldots)$
and thus
$PIP_{KFc} = (1 - k_0) \cdot (\sum_{l=1}^{L} \alpha_l - \sum_{l1 \neq l2} \alpha_{l1}\alpha_{l2} + \sum_{l1 \neq l2 \neq l3} \alpha_{l1}\alpha_{l2}\alpha_{l3} - \ldots)$ …**(a)**
Whereas sorting PIP_{adj} results in:

$$PIP_{adj} = 1 - \{1 - (1 - k_0)\sum_{l=1}^{L} \alpha_l + (1 - k_0)^2 \sum_{l1 \neq l2} \alpha_{l1}\alpha_{l2} - (1 - k_0)^3 \sum_{l1 \neq l2 \neq l3} \alpha_{l1}\alpha_{l2}\alpha_{l3} + \ldots\}$$

$= (1 - k_0) \cdot (\sum_{l=1}^{L} \alpha_l - (1 - k_0)\sum_{l1 \neq l2} \alpha_{l1}\alpha_{l2} + (1 - k_0)^2 \sum_{l1 \neq l2 \neq l3} \alpha_{l1}\alpha_{l2}\alpha_{l3} - \ldots\}$ …**(b)**

(Here … denotes the higher order terms).

In clear cases where there is only one significant (>>0) α for a variant v, letting the significant single effect scaler corresponding to v be $\alpha_{sig}(v)$, according to (a) and (b), $PIP_{KFc}(v) \approx (1 - k_0) \cdot \alpha_{sig}(v)$ as well as $PIP_{adj}(v) \approx (1 - k_0) \cdot \alpha_{sig}(v)$ holds true, and thus two methods would yield nearly identical output.

In other cases where there are multiple significant αs for a variant, PIP_{adj} is slightly smaller than PIP_{KFc} due to multiplication by $(1 - k_0)^{n-1}$ for the n order term. For example, when there are two significant (>>0) α for a variant v, letting the significant single effect scalers corresponding to the variant v be $\alpha_{sig1}(v), \alpha_{sig2}(v)$, then
$PIP_{KFc}(v) - PIP_{adj}(v) \approx (1 - k_0)k_0\, \alpha_{sig1}(v)\alpha_{sig2}(v) \leq 0.25\alpha_{sig1}(v)\alpha_{sig2}(v) \leq 0.25$.
Thus even in an rare case of >1 significant αs, both nearly equal to 1 and $k_0 \approx 0.5$, the difference between two PIP outputs is no more than 0.25.

Although validation is outside of the scope of this manuscript, we thus assume plugging the estimated null gene probability as the `null_weight` in the SuSiE model should provide nearly identical results as we presented in our manuscript.

CAVIAR: The method[3] assigns non-zero prior probability of 0 causal variants, which is fixed as 1/(#possible causal configurations). We note that this prior probability is typically extremely low (e.g. when there are n variants in a locus, even when setting the maximum number of causal variant to be 2, the prior null gene probability is $(1 + n + \frac{n}{2})^{-1}$), and is constant regardless of the marginal association statistics from the observed data. Although detailed analysis is out of the scope of this manuscript, we assume post-hoc adjustment of output PIPs from CAVIAR as done in KFc results in a slightly deflated PIP due to non-zero prior on null gene probability in the fine-mapping step itself. Same discussion applies for other major methods such as CAVIARBF[4], PAINTOR[5], GUESS[6] where the prior null gene probability is non-zero but is typically small and is not dynamically specified as a function of observed data.

DAP-G: The algorithm[7] contains a step to calculate the locus level posterior probability of being a null gene, after fixing the enrichment parameter α (enrichment of genomic annotations). Following the step, DAP-G applies a thresholding process based on user-defined parameters to achieve desired FDR control level, and then apply the causal configuration inference. The difference compared to our proposed KFc method is that 1. DAP-G does not use knockoff to estimate the null gene probability (and uses functional annotations in the step of null gene probability estimation), and 2. DAP-G uses the estimated null gene probability for thresholding rather than continuous PIP adjustment (which can be less or more conservative compared to KFc, depending on the user-defined threshold).

Overall, even though some methods do allow 0 causal variants in the prior, the idea of flexibly estimating the null gene probability and using it to refine PIPs as in our method is unique. We also note that the idea of adjusting fine-mapping results post-hoc exists (e.g. Hutchinson et al.[8]) but the difference resides in that Hutchinson et al. aims to calibrate the credible set size rather than individual PIPs.

Limitations and possible room for improvement in the knockoff based lFDP estimation

As briefly noted in the discussion section, an important limitation of our proposed method is the quality of lFDP estimation based on knockoff genotypes. Possible improvements over our presented method includes 1. constructing multiple knockoff genotypes to reduce noises, as proposed in He et al.[9], 2. alternative methods to quantify test statistics (although we did not observe a major improvement when using weights from LASSO or simple correlation coefficient instead of minimum p-value). Additional concerns for our knockoff application include 1. Small sample size compared to the typical application range of knockoff, 2. Large window size (1Mb) compared to typical knockoff applications, which we leave as a future work for more investigation. In addition, we note that knockoff genotype construction requires raw genotype data, which might not be readily available in many fine-mapping study settings. Although outside of the scope of this manuscript, we believe gene-level local FDP estimation using methods other than knockoffs, such as lFDP estimation from q-value[10] is also valuable, as discussed more in the next section.

Possible alternative fine-mapping models to take null gene probability into account

We note there are possible methods to flexibly take into account the probability of no causal variant in a locus, other than utilizing knockoff as done in our work.

First is a fully Bayesian approach, as presented in e.g. Flutre et al.[11] or Wen et al.[7] Their method averages over the Bayes factors for n cases (n: number of variants in a locus) assuming exact one causal variant in a locus:

$$BF_v = \frac{p(Data \mid v\ is\ the\ only\ causal\ variant\ in\ the\ locus)}{p(Data \mid no\ causal\ variant\ in\ the\ locus)}$$

$$BF_{gene} = \sum_{v=1}^{n} BF_v \,/\, n$$

and turns the gene level Bayes factor into FDR by first obtaining p-values through permutation and then turning into q-values. Conversion of q-values to local FDP estimate can be done as described in e.g. [10]. Although intuitively clear, the limitation of this method includes (1) non-trivial choice of priors when calculating the Bayes factors, (2) non-trivial choice of parameter λ when calculating q-values, and (3) Simplified assumption of exact one causal variant in a locus.

Second is a post-hoc adjustment method based on the PIP distribution, as done in Hutchinson et al.[8] (where they focused on credible set size adjustment rather than global PIP calibration). The method, again restricting the model to have exactly one variant in a locus, first perform standard fine-mapping and then estimates the true causal effect in the unit of |z-score| by weighting the absolute z-scores over all the variants with non-trivial PIPs in a locus:
$\hat{\mu} = \sum_{i \in V} |Z_i| \cdot PIP_i$, where V denotes the set of variants with $PIP > threshold$,
then re-sample marginal z-scores based on $\hat{\mu}$ and the LD matrix, and use the results to adjust credible set estimates. Although their implementation does not directly try to adjust individual PIPs, we envision the re-sampling scheme they presented could be useful for null gene probability estimation (e.g. the $\hat{\mu}$ can be directly turned into p-value, q-value and lFDP), although we note that this approach also suffers from manual choice of thresholds as well as single causal variant assumption.

Third is to reflect the null gene probability at the prior effect sizes. For example, Walters et al.[12] showed using a Laplace prior instead of typical normal priors could increase the accuracy of fine-mapping in the case of a single causal variant in a certain evaluation metric. In line with such approaches, allowing higher density on $\beta = 0$ in the effect size prior and thresholding the variant-level PIP with a certain effect size threshold could result in a reduced number of false discoveries.

Possible application to complex traits or trans-eQTL discovery

Although we restricted our analysis to cis-eQTL fine-mapping, we envision extension of the KFc method to trans-eQTLs or complex traits with various polygenic causal architectures to be relatively simple.

As an example, trans-eQTL discovery could be achieved by quantifying the lFDP for each gene expression phenotype for each 1Mb window instead of just cis-regions (which could reduce the power due to large number of tests, but the lFDP upperbound for individual findings should still be calibrated).

For complex trait fine-mapping, instead of defining a region to fine-map by first finding variants with association p-value passing the threshold (index variant) and then including the variants in LD (passing a user-defined r-squared threshold) of the index variant, one can first divide the genome into non-overlapping window of arbitral size, test the locus level significance and perform fine-mapping for the locus with non-zero lFDP (Which is an idea similar to Sesia et al.[13], although the difference resides in that they try to narrow down the locus as much as possible, whereas we assume applying Bayesian fine-mapping algorithms once the locus level lFDP is defined).

Supplementary Figures

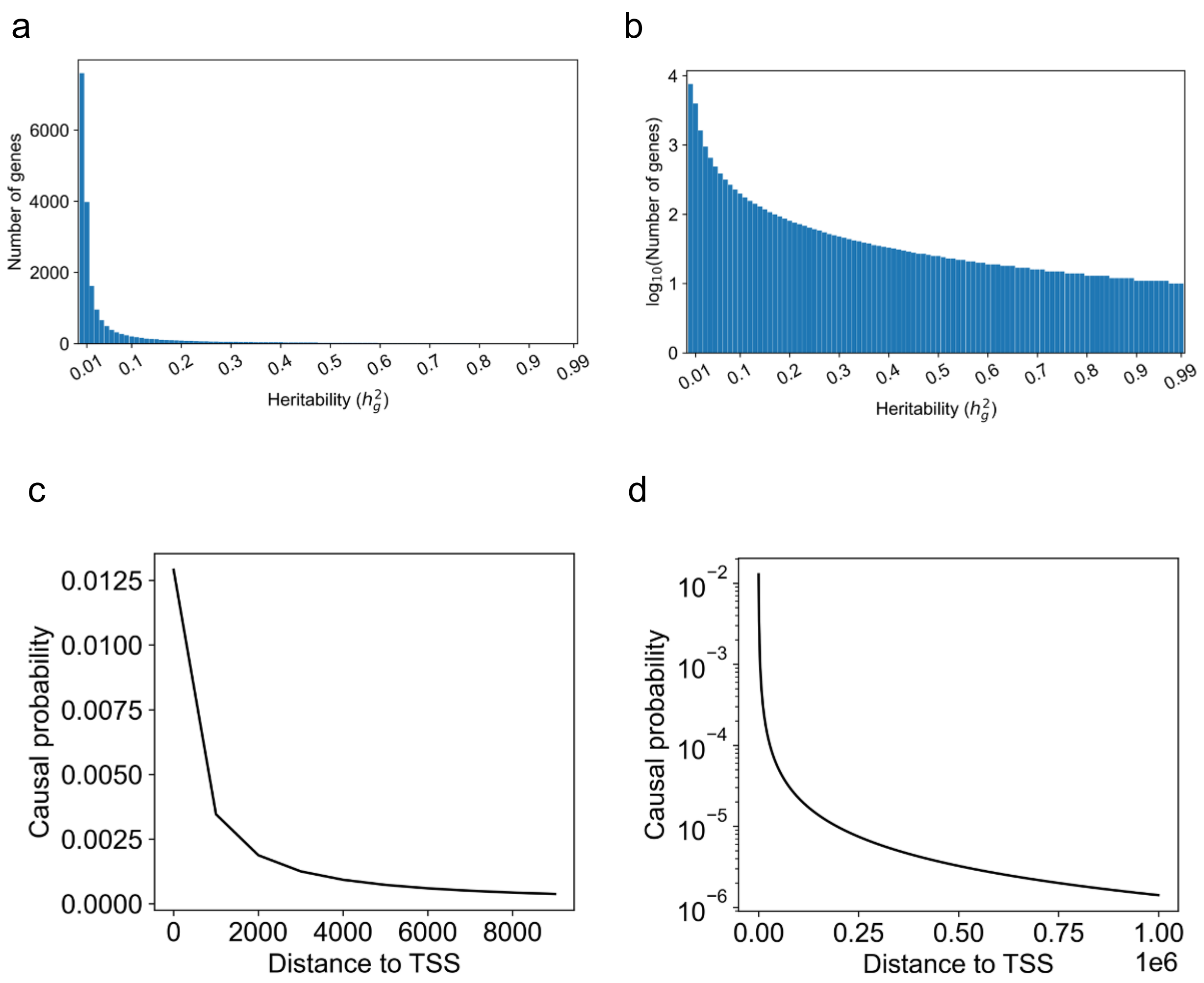

Fig. S1. Details of the simulation setting
a,b. Heritability distribution in our simulation (**b** is in log scale).
c,d. Causal variant distribution as a function of dTSS (**d** is in log scale).

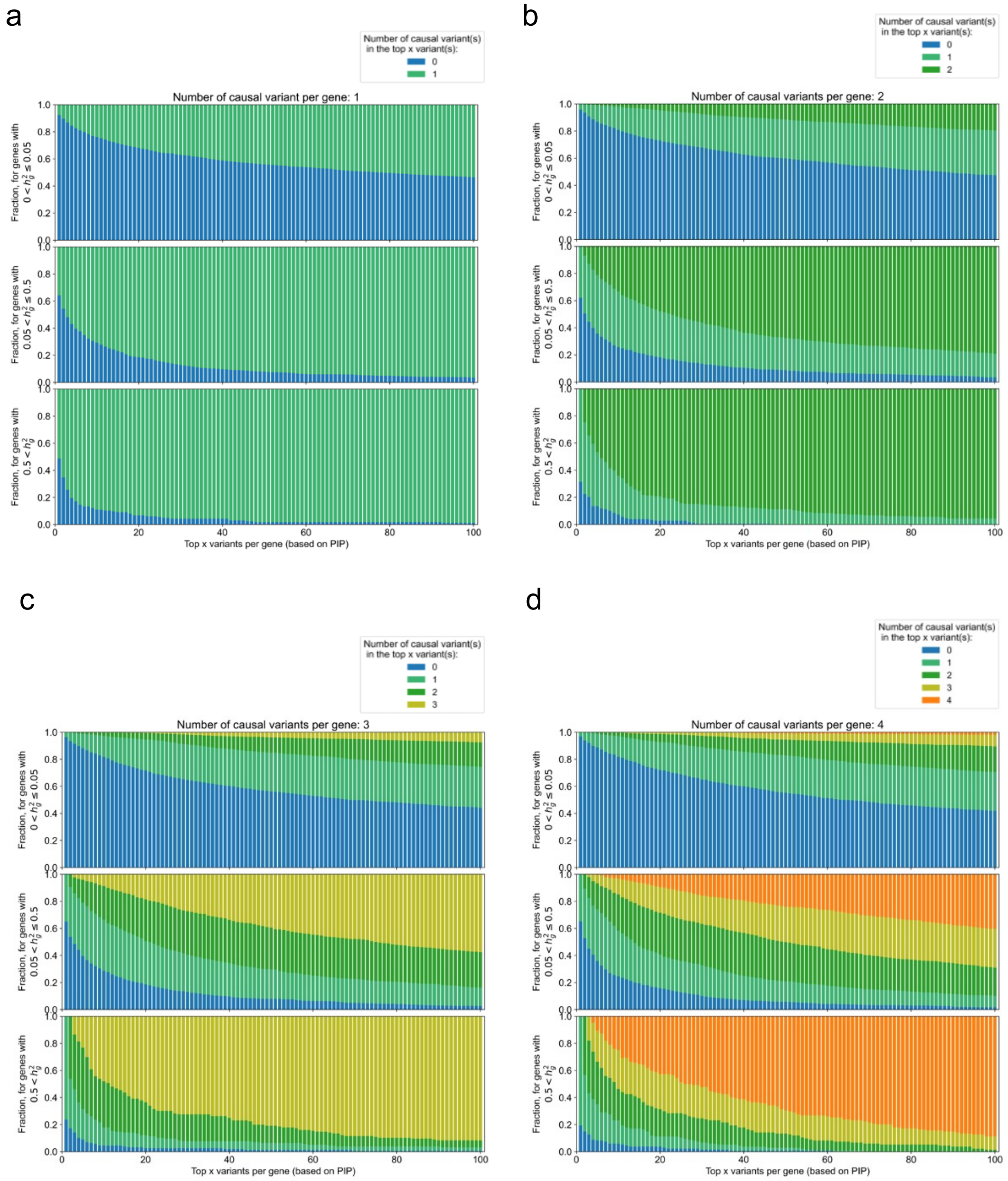
a
Number of causal variant(s) in the top x variant(s):
0
1
Number of causal variant per gene: 1
Fraction, for genes with $0 < h_g^2 \le 0.05$
Fraction, for genes with $0.05 < h_g^2 \le 0.5$
Fraction, for genes with $0.5 < h_g^2$
Top x variants per gene (based on PIP)
b
Number of causal variant(s) in the top x variant(s):
0
1
2
Number of causal variants per gene: 2
Fraction, for genes with $0 < h_g^2 \le 0.05$
Fraction, for genes with $0.05 < h_g^2 \le 0.5$
Fraction, for genes with $0.5 < h_g^2$
Top x variants per gene (based on PIP)
c
Number of causal variant(s) in the top x variant(s):
0
1
2
3
Number of causal variants per gene: 3
Fraction, for genes with $0 < h_g^2 \le 0.05$
Fraction, for genes with $0.05 < h_g^2 \le 0.5$
Fraction, for genes with $0.5 < h_g^2$
Top x variants per gene (based on PIP)
d
Number of causal variant(s) in the top x variant(s):
0
1
2
3
4
Number of causal variants per gene: 4
Fraction, for genes with $0 < h_g^2 \le 0.05$
Fraction, for genes with $0.05 < h_g^2 \le 0.5$
Fraction, for genes with $0.5 < h_g^2$
Top x variants per gene (based on PIP)

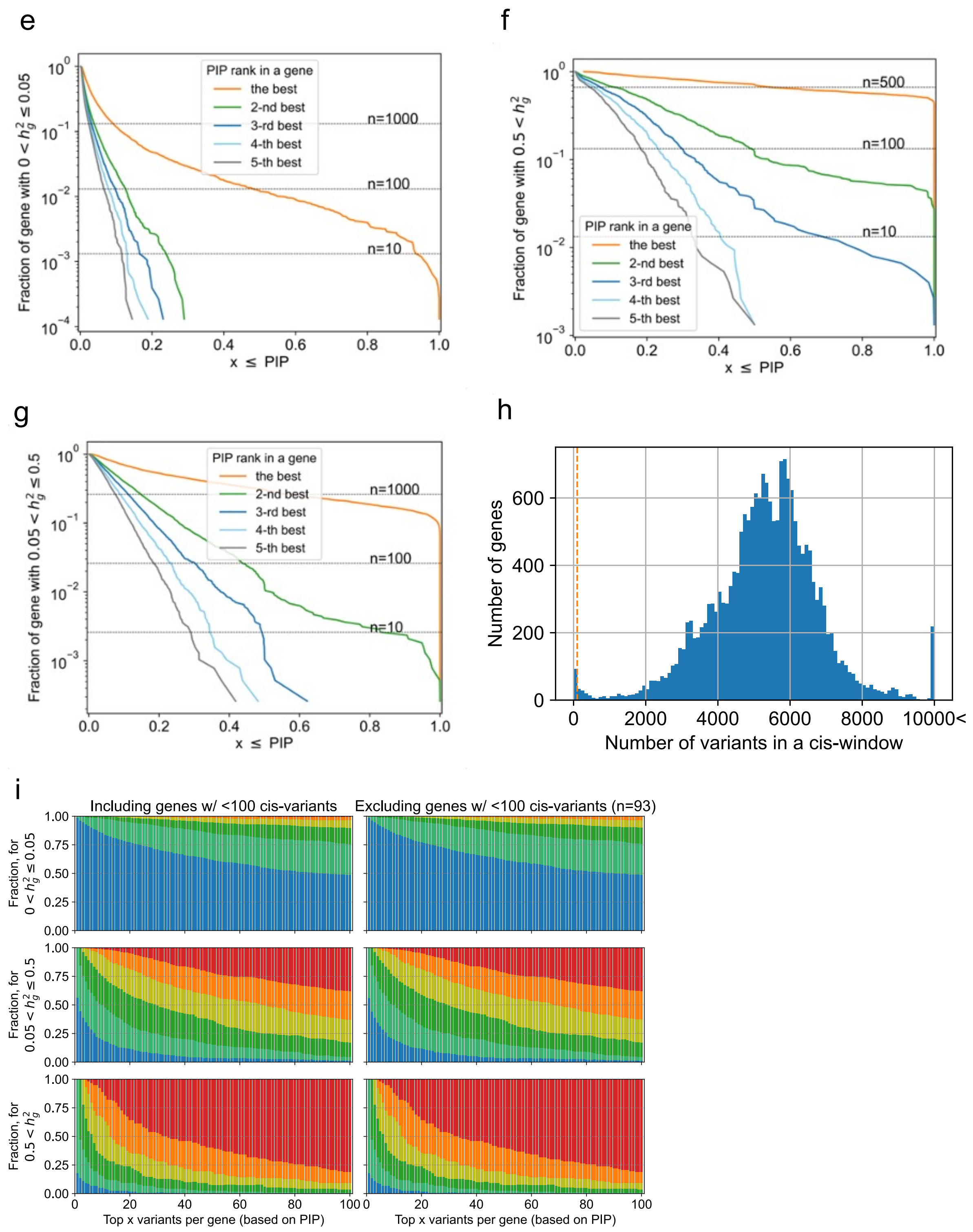

Fig. S2. Evaluation of fine-mapping of the simulation data

a-d. Number of causal variant(s) in the top PIP variant(s) in each gene with varying number of causal variants per gene (**a**:1, **b**:2, **c**:3 and **d**:4 causal variants/gene. The case of 5 causal variants/gene is shown in main **Fig. 2b**).

e-g. Cumulative distribution of top k variants as a function of PIP threshold, in each heritability bin (**e**: low, **f**: middle, **g**: high. The case of null genes is shown in main **Fig. 2c**).

h. Number of variants in a cis-window per gene. The counts are truncated at 10,000 variants for visibility. Orange line corresponds to 100 variants.

i. Removing 93 genes with <100 makes no visible differences in the main **Fig. 2b**.

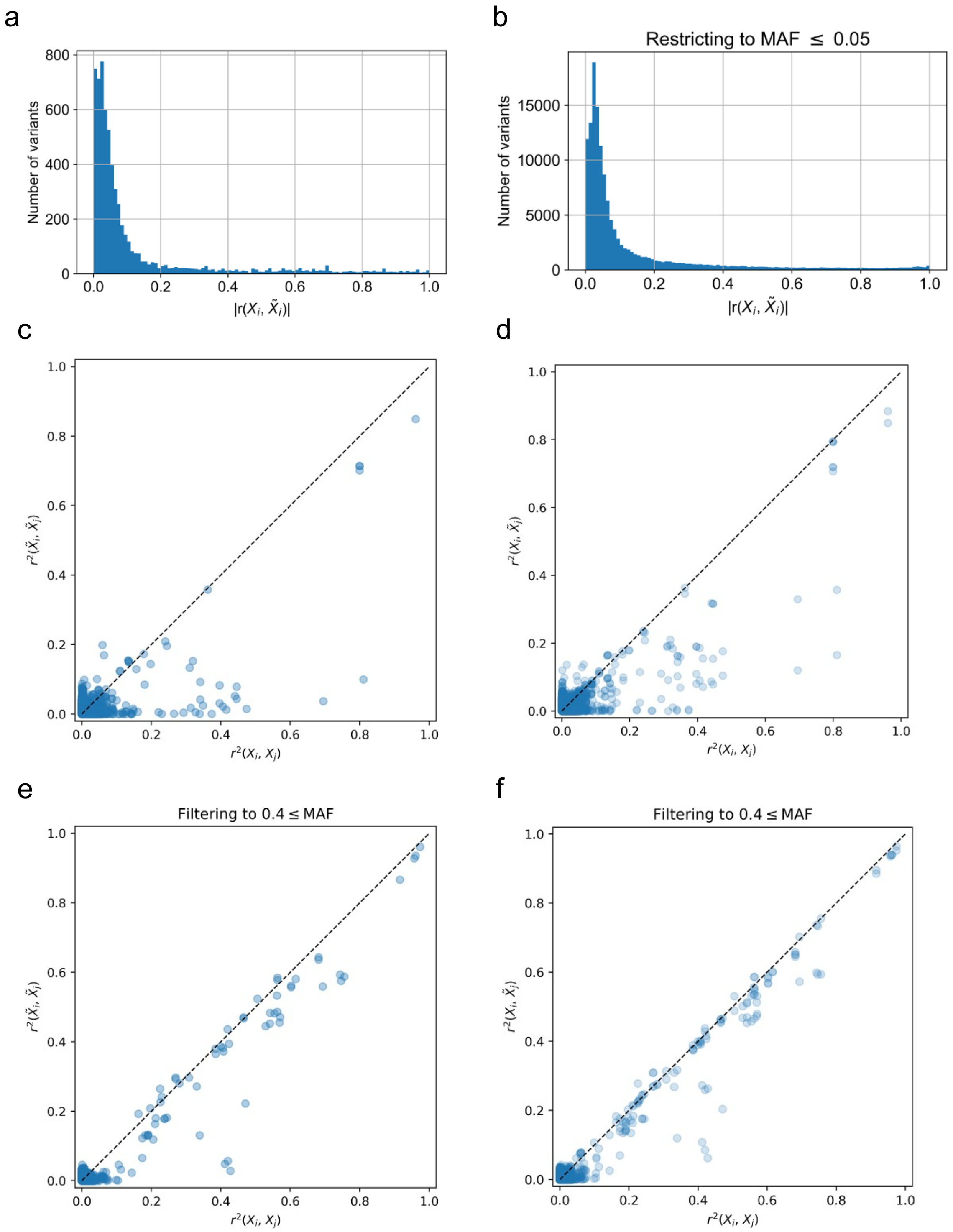
a
Number of variants
|r(X_i, $\tilde{X}_i$)|
b
Restricting to MAF ≤ 0.05
Number of variants
|r(X_i, $\tilde{X}_i$)|
c
$r^2(\tilde{X}_i, \tilde{X}_j)$
$r^2(X_i, X_j)$
d
$r^2(X_i, \tilde{X}_j)$
$r^2(X_i, X_j)$
e
Filtering to 0.4 ≤ MAF
$r^2(\tilde{X}_i, \tilde{X}_j)$
$r^2(X_i, X_j)$
f
Filtering to 0.4 ≤ MAF
$r^2(X_i, \tilde{X}_j)$
$r^2(X_i, X_j)$

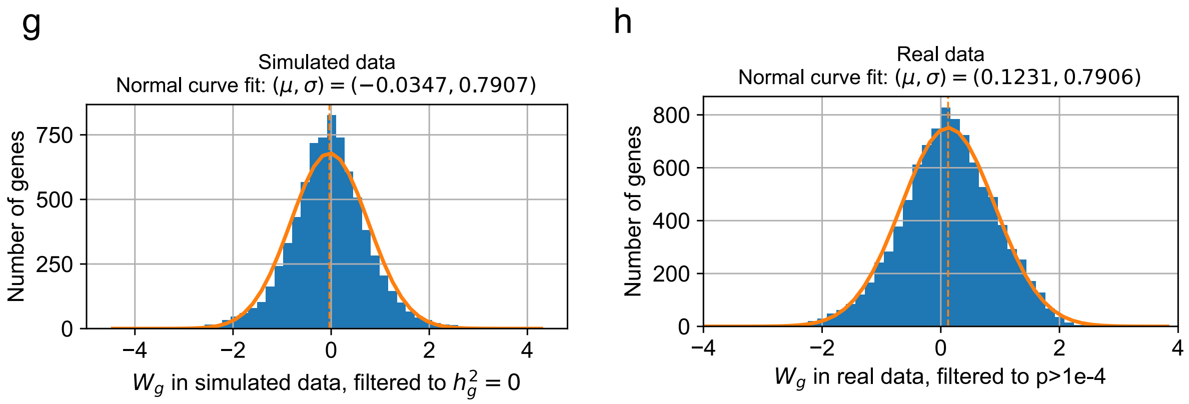

Fig. S3 Knockoff genotype diagnosis

a, b. Distribution of the absolute correlation between the real and corresponding knockoff genotypes for all (**a**) and rare (**b**; MAF≤0.05) variants. The correlation is not high in most cases, validating that the knockoff genotypes are not the identical copy of the real genotypes.

c, d. Scatter plot showing the correlation between real genotypes in different groups (x axis) and the correlation between the corresponding real and knockoff genotypes (y axis) (**c**), or between the corresponding knockoff genotypes (**d**).

e, f. Same as **c** and **d**, restricting to common (0.4≤MAF) variants.

c-f together validates exchangeability of the knockoff genotypes (especially for common variants, as in **e** and **f**). We note that due to large window size (1Mb), correlations are noisy by nature especially for rare variants. 1000 variants from chr22 were randomly sampled in **c** and **d** for computational simplicity.

g, h. The distribution of W_g for null genes in simulation (**g**) and genes with low evidence of genetic regulation (p>1e-4) in real JCTF data (**h**), together with a fitted normal curve. The distribution is near-symmetric and near-normal for null genes, and is slightly shifted towards positive for real data (presumably reflecting the small but non-null regulatory variant effects).

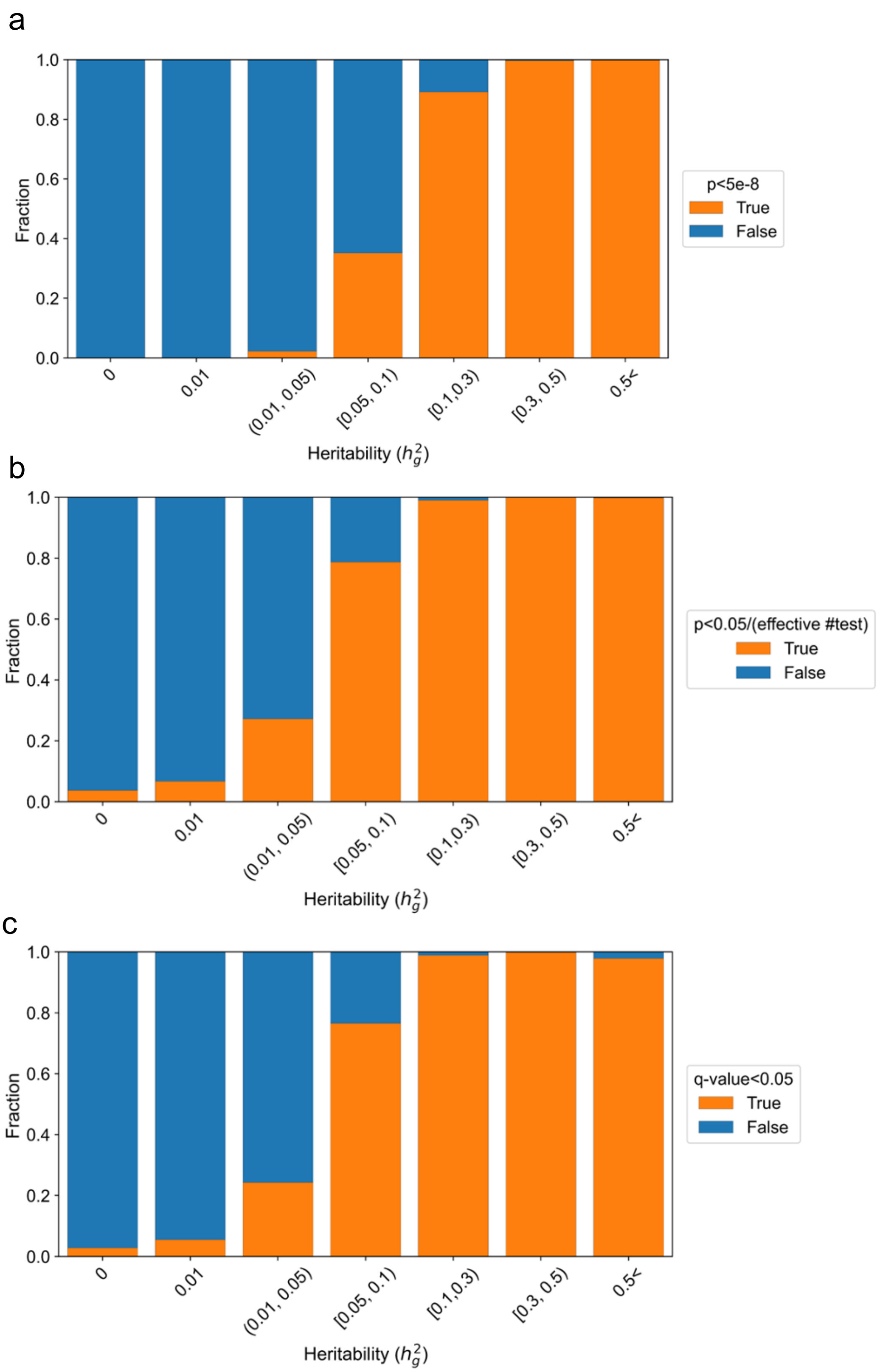

Fig. S4. False and true discovery proportion of different thresholding methods in simulation

X axis is the heritability bin and y axis is the fraction of genes passing (orange) or failing (blue) the threshold, where the threshold is either $p<5e\text{-}8$ (i.e. Bonferroni, **a**), $p<0.05$ divided by the effective number of tests (i.e. EMT, **b**), or q-value<0.05 (i.e. FDR, **c**).

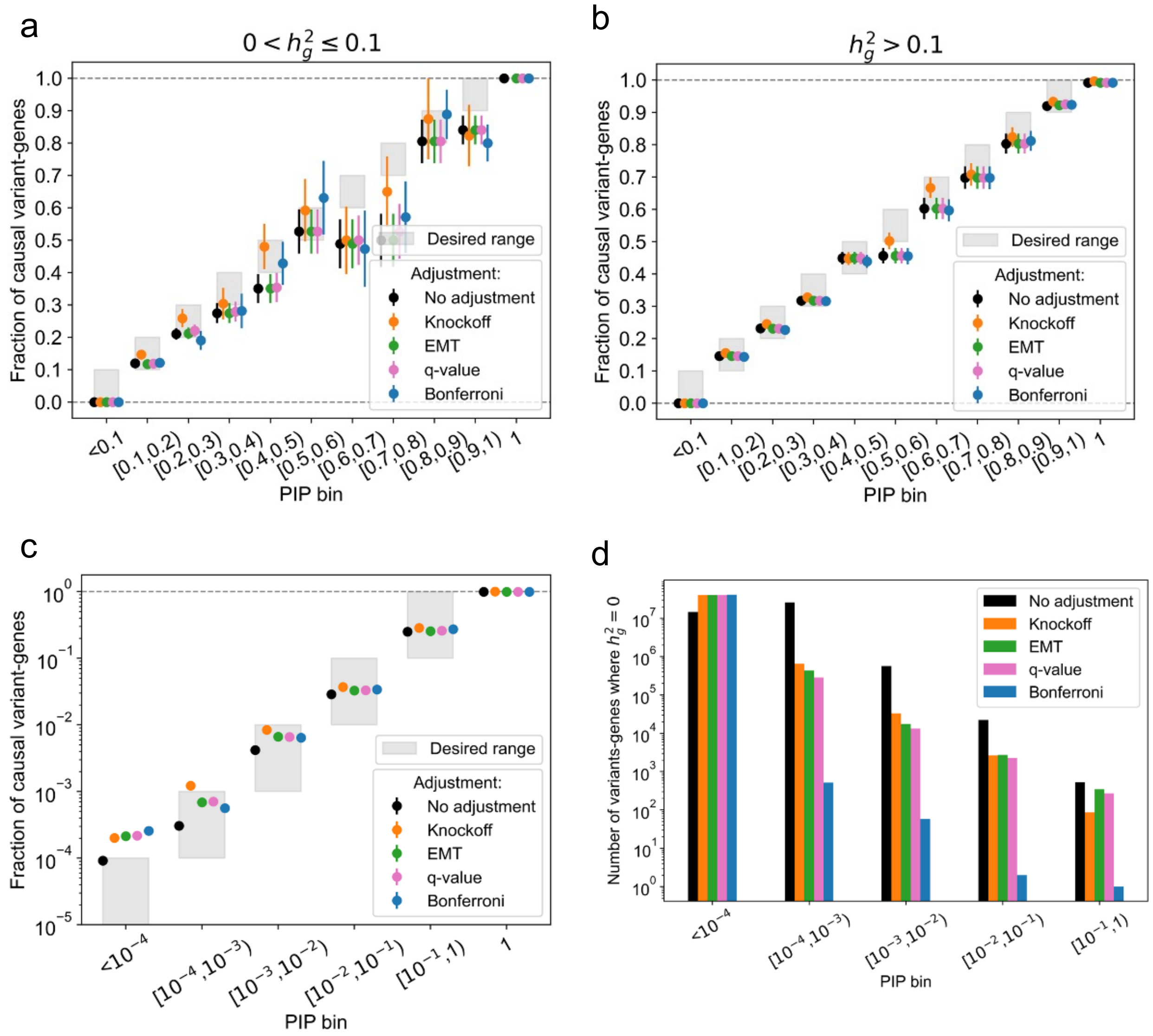

Fig. S5. Detailed inspection of PIP calibration

a, b. Calibration of PIPs for low (**a**) and high (**b**) heritability genes separately.

c. calibration of PIPs in log scale (where KFc does not perform the best in extreme low PIP bins).

d. The number of variants from null genes as a function of PIPs in different methods, where KFc is the 2nd best conservative method following Bonferroni correction.

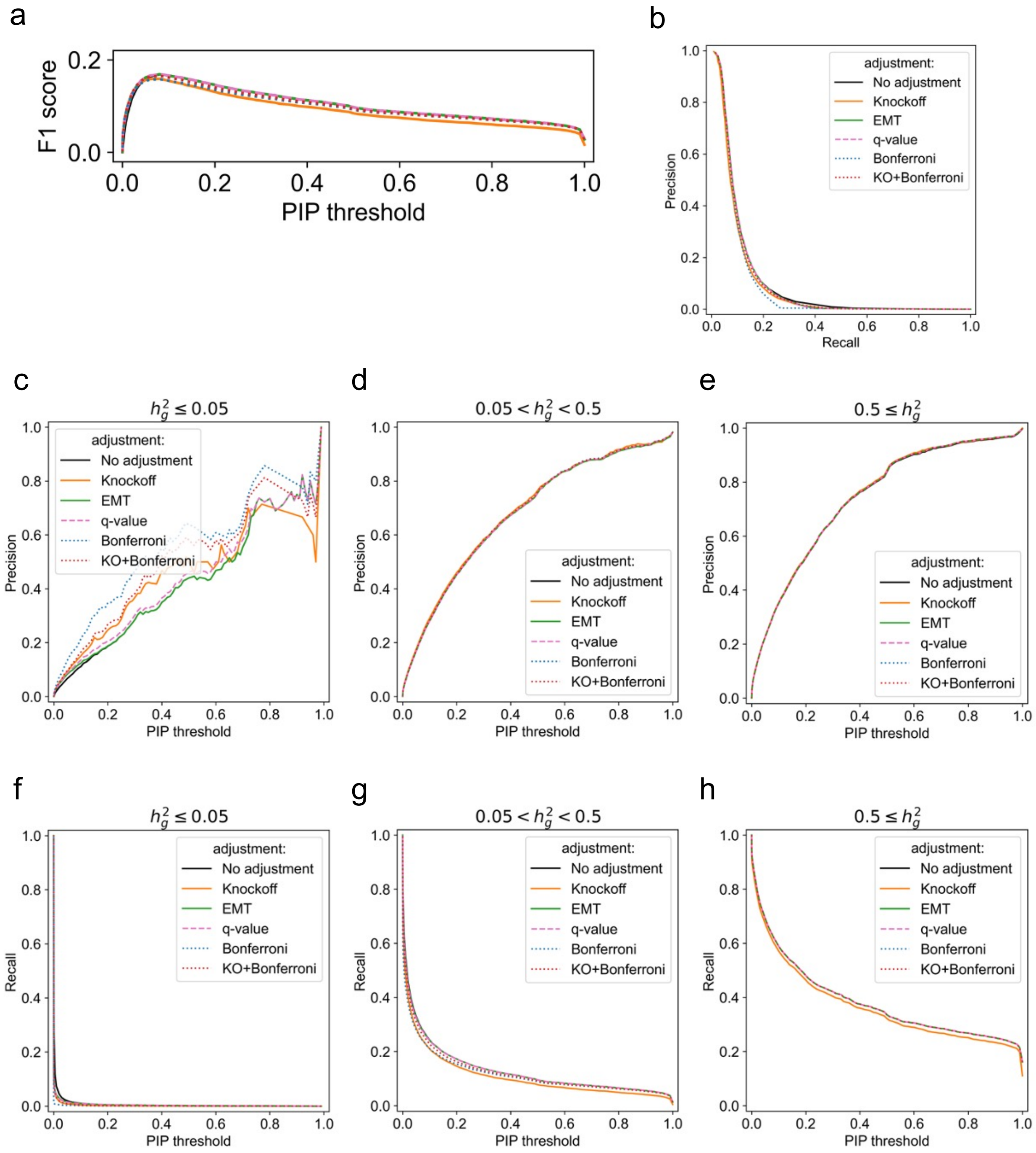
a
F1 score
0.0
0.2
0.0
0.2
0.4
0.6
0.8
1.0
PIP threshold
b
Precision
Recall
adjustment:
No adjustment
Knockoff
EMT
q-value
Bonferroni
KO+Bonferroni
c
$h_g^2 \leq 0.05$
Precision
PIP threshold
d
$0.05 < h_g^2 < 0.5$
Precision
PIP threshold
e
$0.5 \leq h_g^2$
Precision
PIP threshold
f
$h_g^2 \leq 0.05$
Recall
PIP threshold
g
$0.05 < h_g^2 < 0.5$
Recall
PIP threshold
h
$0.5 \leq h_g^2$
Recall
PIP threshold

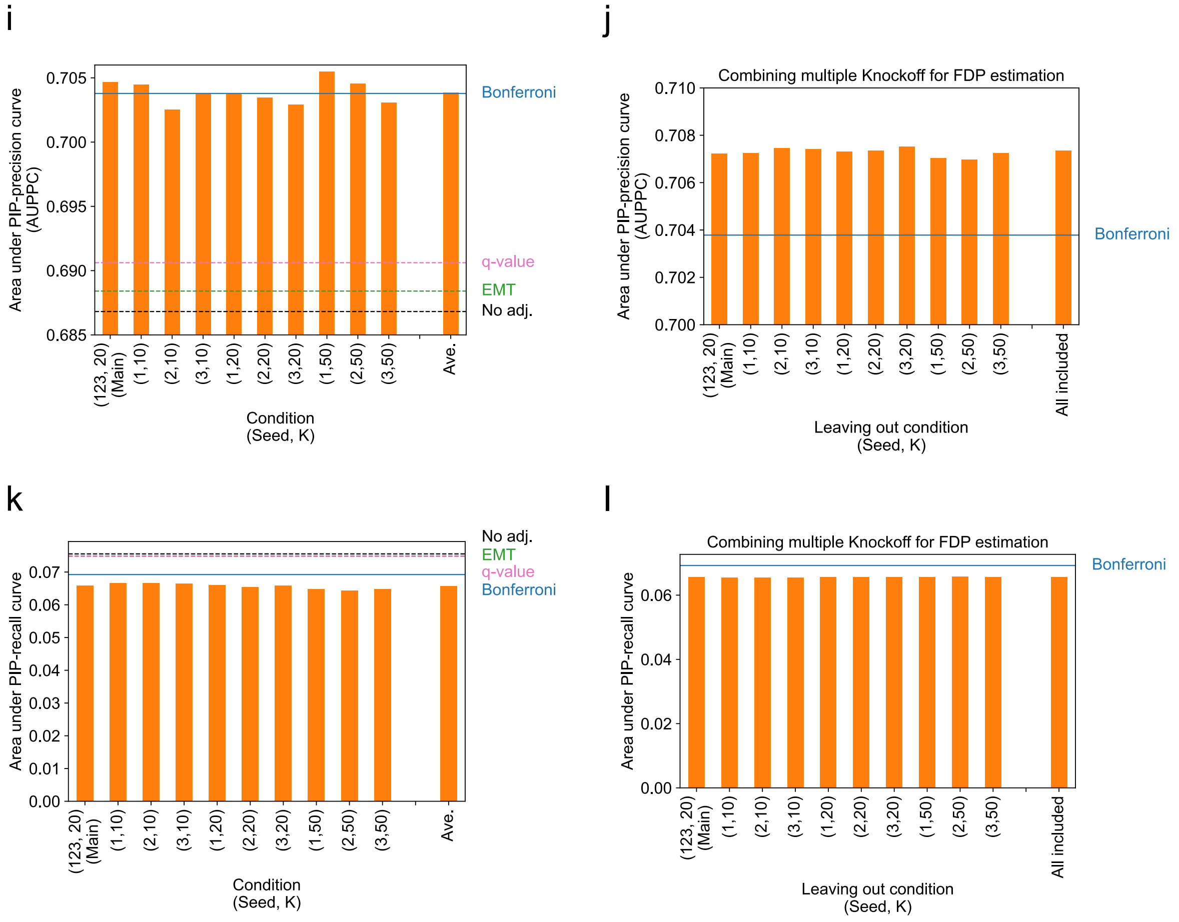

Fig. S6 Detailed inspection of precision and recall of different PIP adjustment methods

a, b. PIP-F1 score curve (**a**) and Precision-recall curve (**b**). Note that although KFc do not perform the best in either metric, these will rarely be the goal of PIP calculation.

c-h. PIP-precision or recall curve stratified by the heritability of genes (**c, f**: low, **d, g**: middle, and **e, h**: high). The differences between the methods are further highlighted for low heritability genes, where the marginal p-value tends to be relatively high and less significant.

i. Area under PIP-precision curve using KFc method with different hyperparameter and seed number setting. In addition to the parameter setting in our main result (seed=123, K=20), additional 3x3 = 9 patterns were tested (seed={1,2,3} times K={10,20,50}, K is the number of clusters in knockoff generation process utilizing fastPHASE).

j. Area under PIP-precision curve comparing the FDP estimation utilizing multiple knockoffs. Combining information from multiple knockoffs consistently present higher precision compared to the Bonferroni method. When all are included, $\widehat{FDP} = \sum_{i=1}^{10} FDP_i \,/\, 10$. When the j-th condition is excluded, $\widehat{FDP} = \sum_{i \neq j} FDP_i \,/\, 9$.

k,l. The corresponding area under PIP-recall curves.

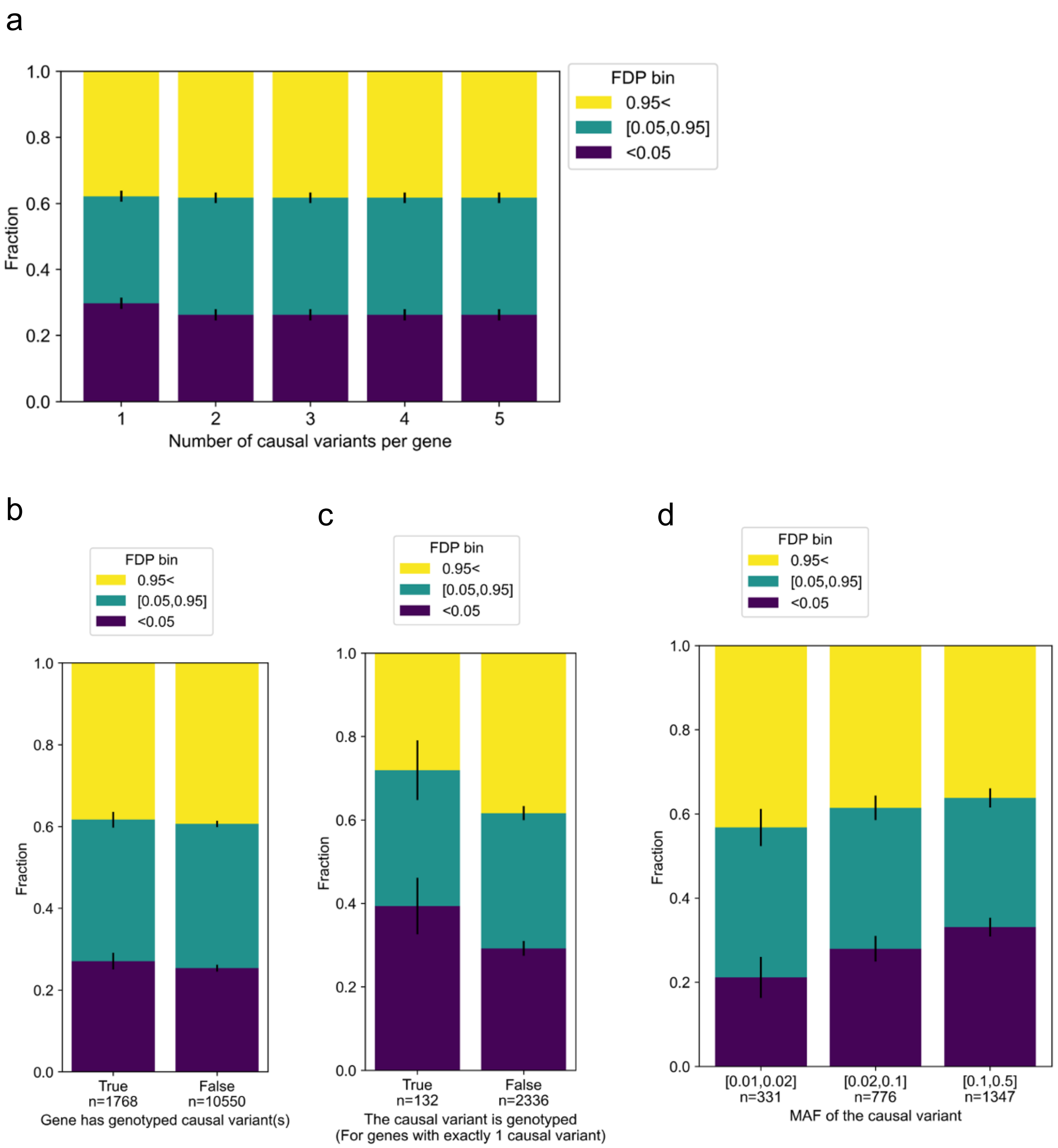

Fig. S7. Characterizing the eGenes with high FDP in simulation
FDP bin distributions for
(**a**) genes with different number of causal variant(s)
(**b**) genes with vs without genotyped causal variant(s)
(**c**) same as (**b**) but restricting to genes with exactly 1 causal variant, and
(**d**) genes with different minor allele frequencies of the causal variant
These observations suggest that FDP estimation becomes hard when the causal variants are rare and/or imputed.

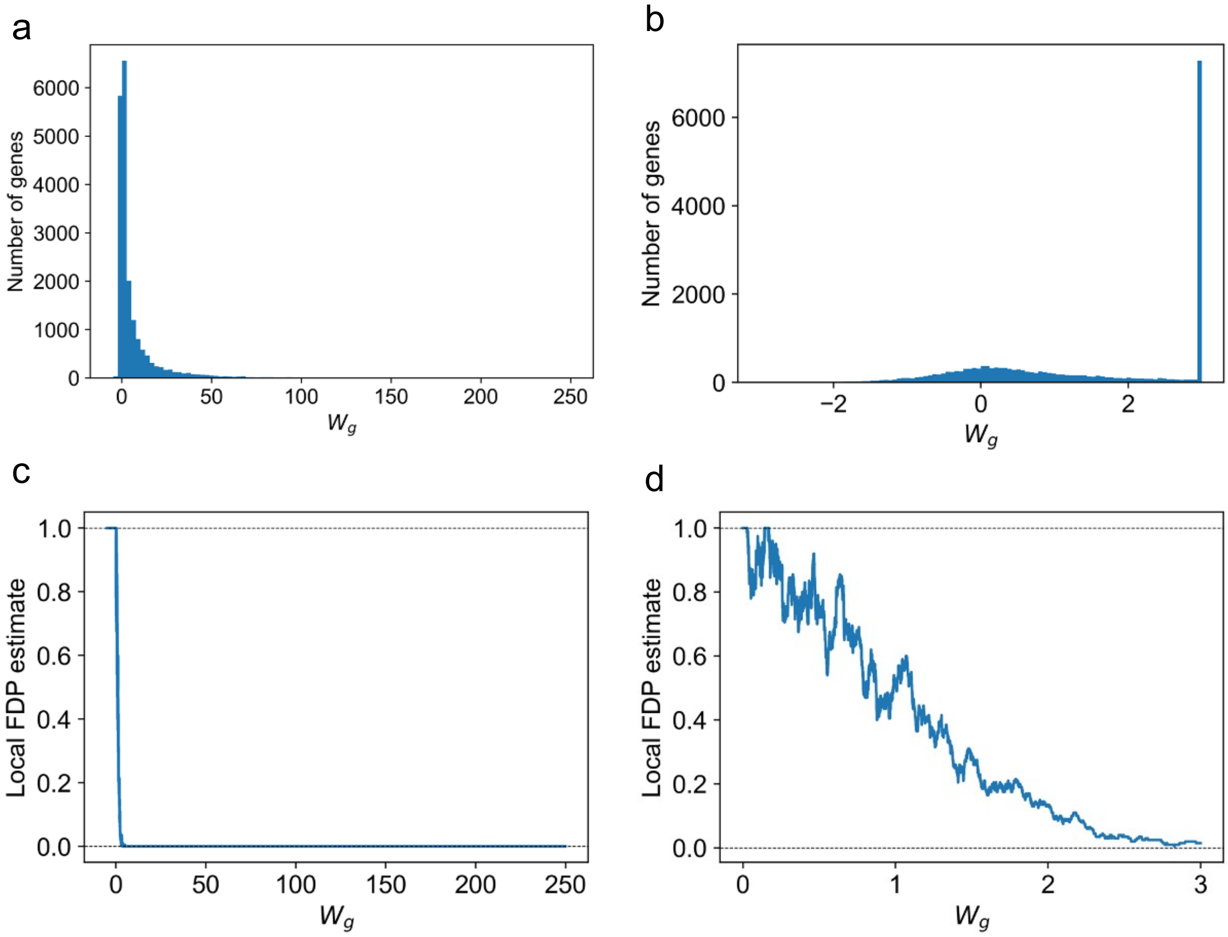

Fig. S8. Details of knockoff construction in real data of JCTF

a, b. Test statistics (W_g) distribution, global (**a**) or truncated at $W_g = 3$ (**b**).

c, d. Local false discovery probability (lFDP) estimation as a function of W_g, global (**c**) or truncated at $W_g = 3$ (**d**).

a

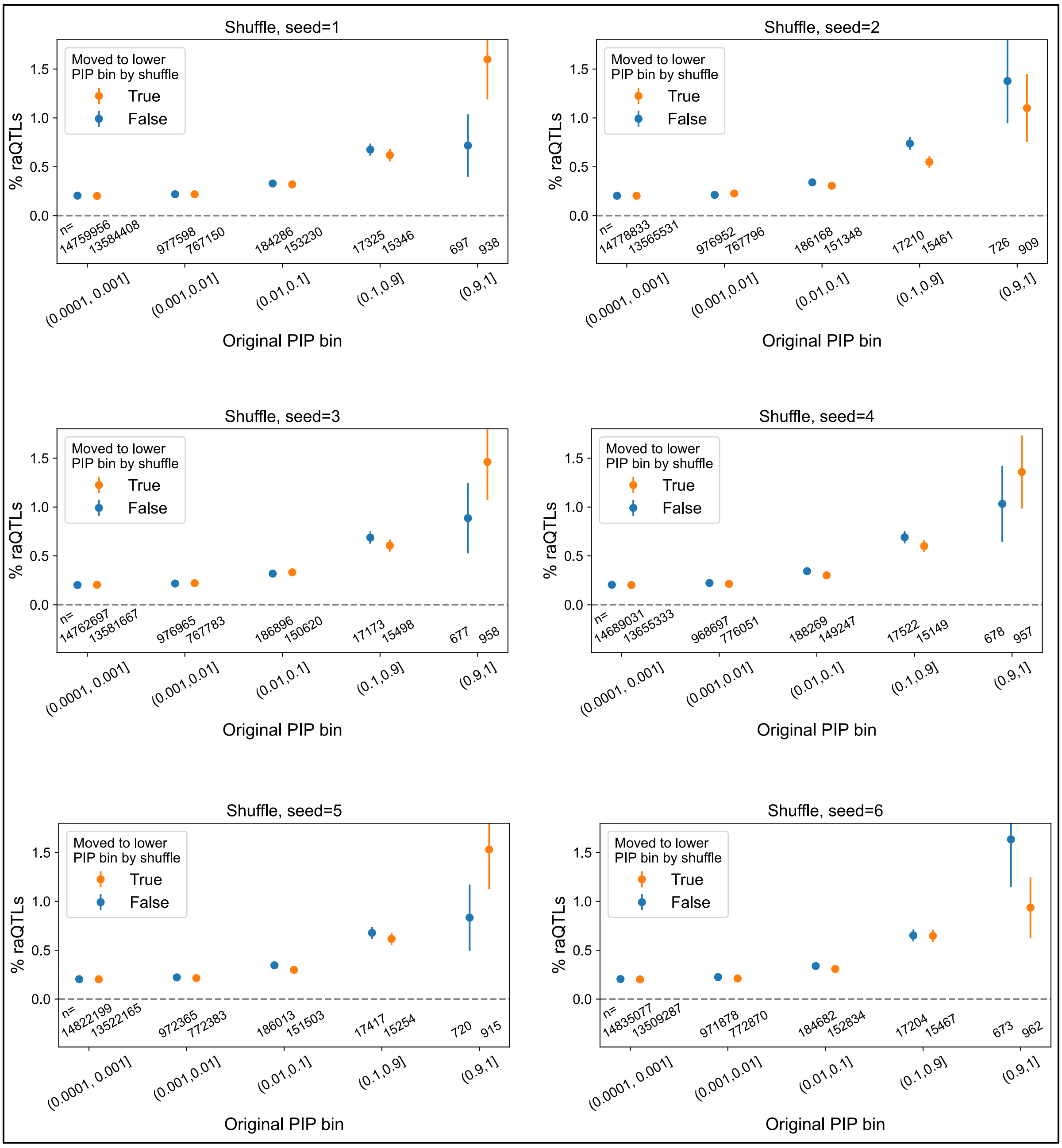
Shuffle, seed=1
Shuffle, seed=2
Shuffle, seed=3
Shuffle, seed=4
Shuffle, seed=5
Shuffle, seed=6
Moved to lower PIP bin by shuffle
True
False
% raQTLs
Original PIP bin
(0.0001, 0.001]
(0.001,0.01]
(0.01,0.1]
(0.1,0.9]
(0.9,1]

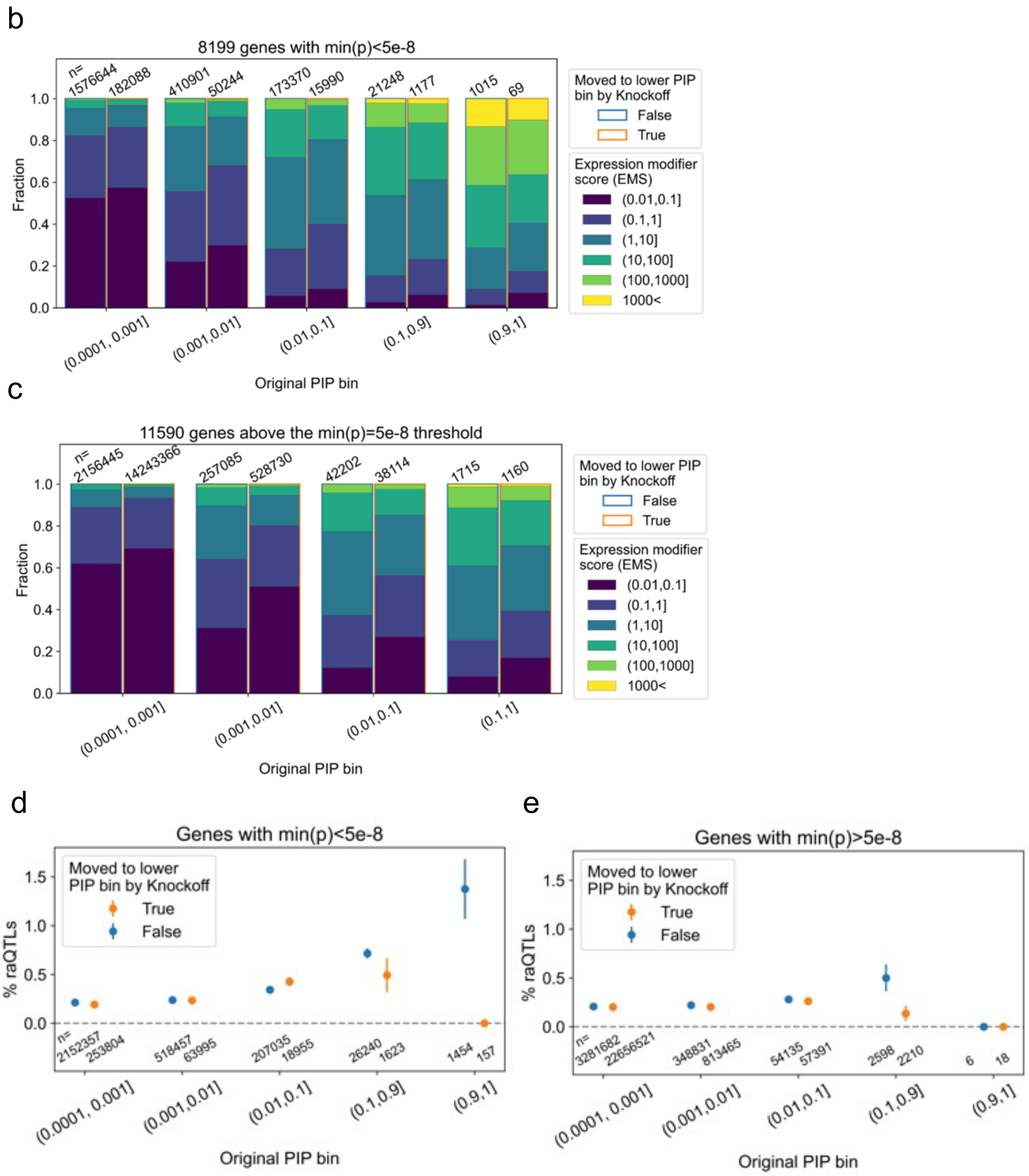

Fig. S9 Distribution of functional hits in various condition

a. Fraction of reporter assay QTL (raQTL) hits when shuffling the FDP assignments for each gene and applying KFc over six different random seeds, as negative controls.

b-d. EMS and raQTL hits before and after KFc adjustment, for different PIP bins, for genes with different minimum p-value (**b.** EMS, p>5e-8 / **c.** EMS, p<5e-8 / **d.** raQTL, p>5e-8 and **e.** raQTL, p<5e-8).The same plot un-stratified for gene minimum p-value are in main **Fig. 5 a** and **b**.

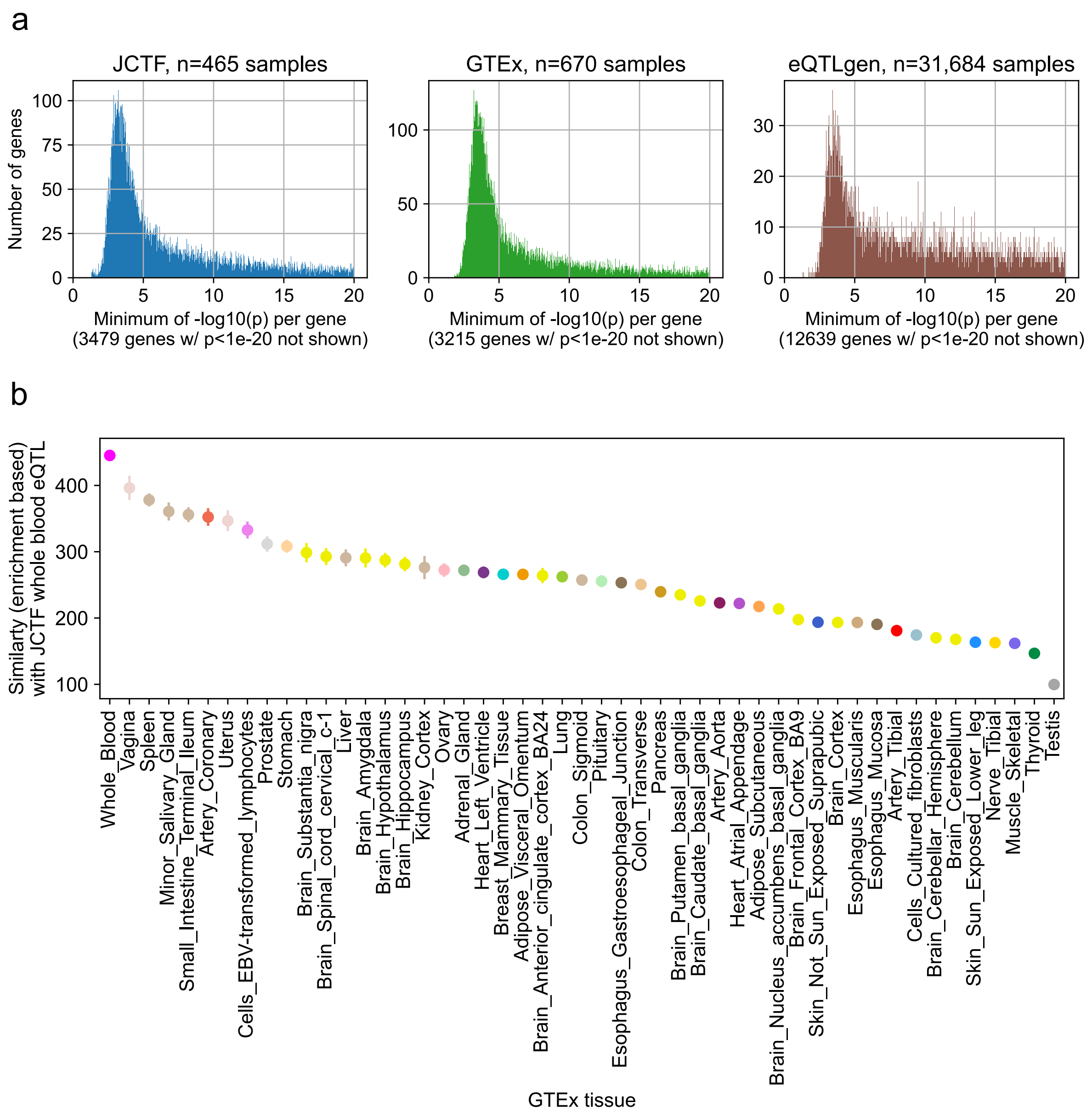

Fig. S10. Comparison between our Japan COVID-19 Task Force (JCTF) and other major blood eQTL datasets

a. Distribution of minimum association p-value per gene in our JCTF dataset, GTEx and eQTLgen. The distribution is continuous at least up to p<1e-20 and includes genes that do not pass the typical Bonferroni threshold (5e-8), in all datasets with different conditions and sample sizes.

b. Similarity of possibly causal variant distribution between JCTF whole blood data and each of GTEx tissues. Similarity was defined as P(PIP>0.1 in GTEx tissue | PIP>0.1 in JCTF whole blood) divided by P(PIP>0.1 in GTEx tissue | any variant-gene in JCTF) and the error bar is the standard error of the numerator divided by the denominator (assuming that the error range in the denominator is relatively small, due to much larger number of variant-genes). The denominator works to control the power differences between GTEx tissues due to sample sizes, data quality and other factors.

Supplementary References

1. Benner, C. *et al.* FINEMAP: efficient variable selection using summary data from genome-wide association studies. *Bioinformatics* **32**, 1493–1501 (2016).
2. Wang, G., Sarkar, A., Carbonetto, P. & Stephens, M. A simple new approach to variable selection in regression, with application to genetic fine mapping. *J. R. Stat. Soc. Ser. B Stat. Methodol.* **82**, 1273–1300 (2020).
3. Hormozdiari, F., Kostem, E., Kang, E. Y., Pasaniuc, B. & Eskin, E. Identifying Causal Variants at Loci with Multiple Signals of Association. *Genetics* **198**, 497–508 (2014).
4. Chen, W. *et al.* Fine Mapping Causal Variants with an Approximate Bayesian Method Using Marginal Test Statistics. *Genetics* **200**, 719–736 (2015).
5. Kichaev, G. *et al.* Integrating Functional Data to Prioritize Causal Variants in Statistical Fine-Mapping Studies. *PLOS Genet.* **10**, e1004722 (2014).
6. Wallace, C. *et al.* Dissection of a Complex Disease Susceptibility Region Using a Bayesian Stochastic Search Approach to Fine Mapping. *PLOS Genet.* **11**, e1005272 (2015).
7. Wen, X., Lee, Y., Luca, F. & Pique-Regi, R. Efficient Integrative Multi-SNP Association Analysis via Deterministic Approximation of Posteriors. *Am. J. Hum. Genet.* **98**, 1114–1129 (2016).
8. Hutchinson, A., Watson, H. & Wallace, C. Improving the coverage of credible sets in Bayesian genetic fine-mapping. *PLOS Comput. Biol.* **16**, e1007829 (2020).
9. He, Z. *et al.* Identification of putative causal loci in whole-genome sequencing data via knockoff statistics. *Nat. Commun.* **12**, 3152 (2021).
10. Strimmer, K. A unified approach to false discovery rate estimation. *BMC Bioinformatics* **9**, 303 (2008).
11. Flutre, T., Wen, X., Pritchard, J. & Stephens, M. A Statistical Framework for Joint eQTL Analysis in Multiple Tissues. *PLOS Genet.* **9**, e1003486 (2013).
12. Walters, K., Cox, A. & Yaacob, H. The utility of the Laplace effect size prior distribution in Bayesian fine-mapping studies. *Genet. Epidemiol.* **45**, 386–401 (2021).
13. Sesia, M., Katsevich, E., Bates, S., Candès, E. & Sabatti, C. Multi-resolution localization of causal variants across the genome. *Nat. Commun.* **11**, 1093 (2020).
